# ECG-Based Emotion Recognition Using Beat-Level Complementary Feature Fusion

**DOI:** 10.3390/s26196169

**Published:** 2026-09-29

**Authors:** Guandi Peng, Ying Guo

**Affiliations:** School of Information Science and Engineering, Shenyang University of Technology, Shenyang 110870, China

**Keywords:** emotion recognition, electrocardiogram, beat-level representation, morphology stability, morphology differences, adaptive dilated convolution, subject-independent evaluation

## Abstract

Physiological-signal-based emotion recognition has received attention in human–computer interaction. Electrocardiograms (ECGs) are readily acquired, and short windows contain repeated beat morphology and beat-wise variations that single-window encoding struggles to separate. Multiscale morphology modeling and training-sample diversity remain limited. We therefore propose Complementary Feature Fusion Dual-Path (CFF-DP), an ECG emotion recognition framework using beat-level complementary feature fusion, with three components: (1) a dual-path framework, where the morphology-stable path constructs representative beats with window-adaptive Gaussian weights, while the morphology-difference path combines beat-wise encoding, positional encoding, and additive attention; gated fusion integrates representations; (2) adaptive dilated convolution (ADConv), which extracts multiscale beat-morphology features using shared kernels and input-dependent scale weights; and (3) deviation-based beat-oriented augmentation (DBOA), which adjusts real-noise injection probability and target signal-to-noise ratio according to morphological deviation. CFF-DP achieved 43.39% mean Macro-F1, close to the best comparator, with the fewest multiply–accumulate operations in five-seed WESAD three-class leave-one-subject-out (LOSO) evaluation, although recognition mainly distinguishes stress, with limited amusement discrimination. With short-gap calibration and testing within the same recording, fine-tuning using 40 s per class achieved 76.72% Macro-F1, exceeding six comparators. Binary DREAMER LOSO retained WESAD hyperparameters: valence Macro-F1 exceeded six comparators, whereas arousal fell below four; both remained below uniform random baselines, indicating limited recognition under current experimental conditions. The framework combines computational efficiency with within-record personalization advantages, although cross-subject recognition remains limited.

## 1. Introduction

Emotion recognition aims to enable computing systems to perceive human affective states and has applications in human–computer interaction, health monitoring, and digital mental health [1,2,3,4]. Speech and facial expressions convey overt emotions but are susceptible to voluntary control and environmental conditions. Physiological signals record internal changes during emotion elicitation, providing another basis for objective emotion assessment. Among these signals, the electrocardiogram (ECG) reflects cardiac electrical activity and autonomic regulation and can be continuously acquired using chest straps and wearable devices [5], making it suitable for low-burden monitoring of emotional states.

Traditional methods construct features from heart rate, heart rate variability, and waveform statistics, and then classify them using support vector machines, random forests, or decision trees [6,7]. These features compress ECG into physiologically meaningful statistical descriptions, but local morphology within each beat still needs to be retained through waveform encoding. To reduce reliance on handcrafted features, researchers have introduced deep learning into ECG emotion recognition. Nita et al. [8] combined data augmentation with a convolutional neural network (CNN) to learn classification representations from expanded training samples. Dar et al. [9] combined a one-dimensional CNN with a long short-term memory (LSTM) network, using convolution to extract local waveforms before recurrent units learned sequential relationships. Convolutional and recurrent structures connect local waveforms with temporal representations, but window-level encoding still needs to carry common morphology and beat-wise variations within the same representation.

Other studies supplement a single waveform representation through multimodal fusion or time–frequency transforms. Wang and Wang [10] extracted time-domain, frequency-domain, and nonlinear features from electroencephalograms (EEGs) and ECGs. After feature selection using a random forest, they used a one-dimensional CNN, a gated recurrent unit (GRU), and attention for joint modeling; this design combines local feature extraction with temporal selection. Kumar et al. [11] used the short-time Fourier transform (STFT) and continuous wavelet transform (CWT) to construct time–frequency representations of physiological signals, allowing convolutional networks to use frequency information at different temporal scales. Related multi-view studies also coordinate local and global dependencies through hierarchical aggregation and assign view weights using sample-level reliability [12,13]. Multimodal and multi-view methods provide ideas for complementary information fusion, whereas single-lead ECG emotion recognition requires complementary features to be organized within the same signal so that common morphology in repeated beats and beat-wise information before aggregation enter separate encoding processes.

An ECG beat consists of structures such as the P wave, QRS complex, ST segment, and T wave, which differ in duration and rate of change. The QRS complex is short and steep, whereas the P and T waves change more gradually, making it difficult for a fixed receptive field to capture both sharp local changes and broad morphology. The random convolutional kernel method expands the scale range of ECG features using one-dimensional kernels of different lengths [14]. Selective Kernel Network (SKNet) selects receptive fields among multiple convolutional branches [15], Conditionally Parameterized Convolution (CondConv) combines expert kernels according to the input [16], and Dynamic Convolution aggregates parallel kernels through attention [17]. Kernel-Sharing Parallel Atrous Convolution (KPAC) shares kernels among parallel dilated branches and fuses their responses using scale attention [18]. For ECG emotion recognition, the Spatially Enhanced Pyramid Split Attention convolutional neural network (SEPSA-CNN) uses dynamic time warping (DTW) to remove morphological outlier beats and combines multiscale grouped convolution, spatial–channel attention, and self-gating to form representations [19]. Building on these studies, we retain all valid beats and separately encode the aggregated representative morphology and the beat-wise morphology before aggregation. We also design adaptive dilated convolution (ADConv) with shared kernels, allowing weights to be assigned to the same set of beat morphologies across dilation scales according to the input.

ECG acquisition is also affected by muscle activity, electrode motion, and wearing conditions. Data augmentation has been used for ECG emotion recognition and ECG analysis [20,21,22], but common segment-level perturbations do not distinguish the morphological states of different beats within a window. Bandpass filtering suppresses baseline drift and some high-frequency components, but muscle artifacts overlap with the ECG spectrum, making in-band muscle artifacts difficult to eliminate through low-pass filtering [23]. Motion-induced changes at the electrode–skin interface also produce electrode motion artifacts [24]. The MIT-BIH Noise Stress Test Database (NSTDB) contains muscle artifact (ma) and electrode motion artifact (em) recordings [25] that can be used to construct perturbations resembling acquisition conditions. Previous single-lead ECG research has shown that introducing multiple noise types and signal-to-noise ratios during training can improve adaptation to noisy conditions [26]. We therefore construct beat-wise augmentation using real NSTDB noise and adjust its probability and strength according to each beat’s deviation from the representative morphology.

In summary, single-lead ECG emotion recognition still faces three key problems. First, a 5 s ECG window contains both repeated common P–QRS–T morphology and local variations retained in individual beats before aggregation. Direct encoding of the complete segment through a single path makes it difficult to fully use the complementarity of these two types of information. Second, waveform structures correspond to different temporal scales. Assigning independent kernels to each scale increases the parameter count, whereas fixed scales have difficulty adapting to changes in input morphology. Finally, uniform segment-level augmentation does not consider each beat’s deviation from the representative morphology within the window and may cover the original beat-wise differences with the same perturbation. To address these problems, we propose the Complementary Feature Fusion Dual-Path (CFF-DP) framework, which first constructs beat sequences anchored at R peaks and then extracts complementary features through two paths. The Morphology-Stable (MS) path aggregates valid beats into a representative beat using weights and extracts multiscale common morphology through an Adaptive Dilated Network (ADNet) composed of stacked Adaptive Dilated Blocks (ADBlocks). The Morphology-Difference (MD) path uses a shared one-dimensional CNN and bidirectional gated recurrent unit (BiGRU) to encode each beat, combining positional encoding with additive attention to retain beat-wise morphology and relative order before aggregation. The two types of features are fused through a gated multimodal unit (GMU) [27] and a residual multilayer perceptron (MLP), followed by a fusion MLP and classification head for emotion recognition. To balance scale adaptability for common morphology with parameter cost, ADConv in the MS path extracts features at multiple dilation rates using shared depthwise kernels and input-dependent scale weights. To increase beat-wise training-sample diversity, Deviation-Based Beat-Oriented Augmentation (DBOA) uses real muscle and electrode motion noise to adjust the perturbation applied to each beat according to morphological deviation. On the main dataset, the experiments use leave-one-subject-out (LOSO) evaluation outside a fixed validation cohort, keeping model selection independent of the test subjects, and examine short-term fine-tuning for target subjects within the same framework.

To examine the applicability of the established training settings under another emotion-elicitation condition, we further conduct supplementary binary LOSO experiments on valence and arousal on another dataset and analyze beat-level complementary representations across the two affective dimensions.

The contributions of this study are as follows:

- We propose the CFF-DP dual-path framework to separately encode aggregated representative morphology and beat-wise morphology before aggregation within the same 5 s ECG window and perform complementary fusion through a gating unit and residual mapping.
- We design ADConv for one-dimensional ECG signals, reusing the same set of depthwise kernels at multiple dilation rates and fusing branch responses with input-dependent scale weights, so that weights can be assigned to beat morphology across dilation scales according to the input.
- We propose DBOA, which samples ma, em, and mixed noise from NSTDB online and jointly sets the augmentation probability and target signal-to-noise ratio (SNR) according to each beat’s normalized morphological deviation from the representative beat.
- On the main dataset, we establish an evaluation protocol with mutually exclusive validation subjects and 12 test subjects, including 12-fold leave-one-subject-out testing and continuous-time personalized fine-tuning. Macro-averaged F1 (Macro-F1) is the primary evaluation metric, with accuracy (ACC) as a supplementary metric; five-seed evaluation is used to report mean performance, between-subject differences, and seed variability. We further conduct a 20-fold binary LOSO evaluation of valence and arousal on the DREAMER dataset to examine the applicability of the established training settings across affective dimensions.

## 2. Materials and Methods

### 2.1. Materials

#### 2.1.1. WESAD Dataset and Task Definition

The Wearable Stress and Affect Detection (WESAD) dataset is a public multimodal dataset acquired under controlled laboratory conditions [28]. The original study involved 17 participants. Recordings from S1 and S12 were excluded because of sensor malfunction, leaving data from 15 participants, including 12 men and 3 women, with a mean age of 27.5 ± 2.4 years. The chest-worn RespiBAN Professional synchronously recorded ECG, electrodermal activity, electromyography, respiration, body temperature, and triaxial acceleration at 700 Hz. The wrist-worn Empatica E4 recorded blood volume pulse, electrodermal activity, body temperature, and acceleration. We use the chest single-lead ECG as the model input. Table 1 summarizes the data, labels, and evaluation settings.

To elicit neutral, stress, and amusement states, the original experiment included baseline recording, emotional stimulation, and recovery. The baseline phase lasted 20 min, during which participants read neutral materials while sitting or standing. The amusement phase involved watching 11 funny video clips totaling 392 s. The stress phase used the Trier Social Stress Test, consisting of public speaking and mental arithmetic tasks lasting 5 min each. The order of the stress and amusement phases alternated across participants, and guided meditation followed both types of stimulation to support emotional recovery. The original study also used affect, anxiety, and self-assessment scales to check the stimulation effects, while the three-class labels were determined by the experimental phase. We adopt its three-class Baseline, Stress, and Amusement tasks, corresponding to labels 1, 2, and 3 in the public data. Each continuous state recording is separated according to its original label and divided into 5 s windows within the state. Each window retains the subject identifier, state label, and original time position for subject-independent splitting and continuous-time splitting in personalized experiments.

#### 2.1.2. DREAMER Dataset and Supplementary Tasks

DREAMER elicits physiological responses through emotional video clips and simultaneously records EEG and ECG signals [29]. The original study recruited 25 volunteers; incomplete recordings from two volunteers due to technical problems were excluded, leaving public data from 23 participants, including 14 men and 9 women. Each participant watched 18 clips lasting 65–393 s and rated valence, arousal, and dominance on a 1–5 scale after each clip. ECG was acquired with a Shimmer device at 256 Hz. To standardize the analysis duration and allow time for the target emotion to be elicited, the original study analyzed signals from the last 60 s of each video stimulus. We follow this temporal selection and use the first ECG lead from this interval to additionally assess subject-independent recognition of affective ratings, defining separate binary valence and arousal tasks. A threshold of 2.5 assigns ratings of 1 and 2 to the low class and ratings of 3, 4, and 5 to the high class. Windows within a clip inherit the corresponding rating label assigned to that clip by the participant.

### 2.2. Method Framework

**Figure 1:**
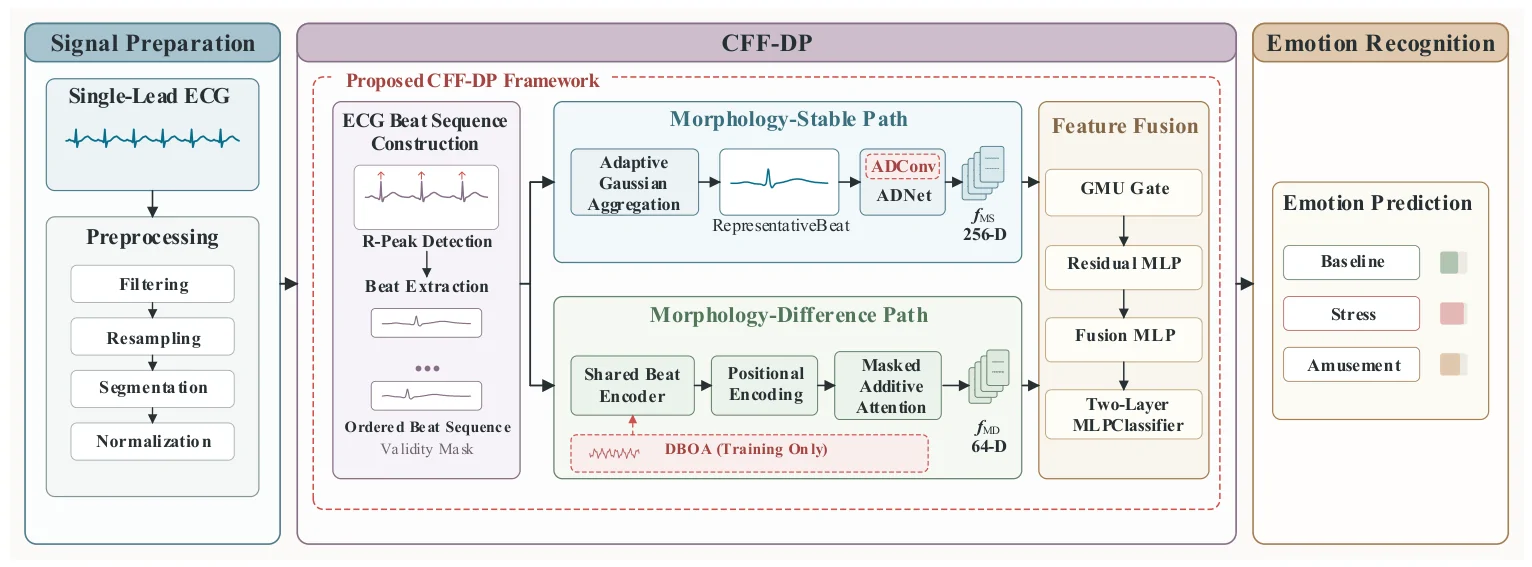
Overall architecture for ECG-based emotion recognition using beat-level complementary feature fusion. The ellipsis denotes additional beats. Abbreviations: CFF-DP, complementary feature fusion dual-path; MS, morphology-stable; MD, morphology-difference; ADConv, adaptive dilated convolution; ADNet, adaptive dilated network; DBOA, deviation-based beat-oriented augmentation.

To retain both common morphology and beat-wise differences from the same 5 s ECG window, we divide the processing pipeline into signal preprocessing, beat sequence construction, dual-path feature extraction, feature fusion, and emotion classification. Figure 1 shows the overall method.

Preprocessing checks for missing samples and sequentially performs bandpass filtering, resampling, window segmentation, and window normalization to obtain standardized 5 s signal windows. CFF-DP then uses the beat sequence construction module to detect R peaks within each window, extract R-peak-aligned beats, and generate a valid-position mask. Two complementary inputs are constructed from the same beat sequence: the MS path aggregates valid beats with Gaussian weights and extracts multiscale common morphology using ADNet containing ADConv; the MD path encodes each valid beat with a shared CNN–BiGRU, adds positional encoding, and summarizes the beat-wise representations through masked additive attention. The two paths are fused through a GMU and residual MLP to produce three-class predictions. During training, DBOA perturbs the valid beats in the MD path, while the representative beat in the MS path remains unchanged.

### 2.3. Preprocessing

**Figure 2:**
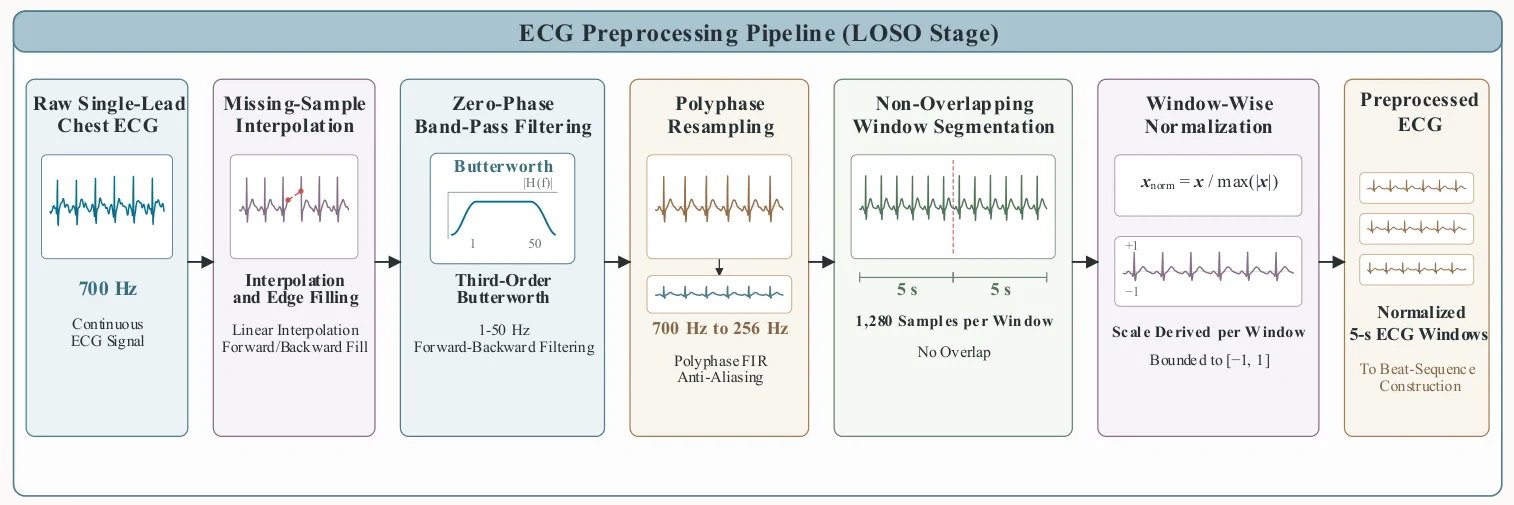
ECG signal preprocessing for LOSO, including filtering, resampling, window segmentation, and normalization.

To provide continuous numerical sequences for filtering and resampling, the signal-level preprocessing in Figure 2 includes a missing-sample check.

If samples are missing within a signal, they are linearly interpolated along the time axis; otherwise, the original samples are retained. Common interference during ECG acquisition includes low-frequency baseline drift, power-line noise near 50 Hz, and muscle noise distributed over a broad middle-to-high-frequency range. To address these components, the WESAD chest ECG is processed with a third-order Butterworth bandpass filter at 1–50 Hz to reduce low-frequency drift and some high-frequency interference while retaining the main P–QRS–T morphology. Because bandpass filtering leaves in-band muscle and electrode motion artifacts that overlap with the ECG spectrum, DBOA uses real-noise augmentation to simulate these possible acquisition disturbances. Polyphase resampling with anti-aliasing then reduces the sampling rate from 700 Hz to 256 Hz, shortening the input and reducing computation while maintaining temporal resolution for beat morphology. Each state recording is subsequently divided into 5 s windows within label boundaries. To reduce amplitude-scale differences caused by subjects and wearing conditions, we normalize each window by its own maximum absolute value so that amplitude scaling depends only on the current window. Let the window signal be *x*. Normalization is calculated using Equation (1), scaling the amplitude range to [−1, 1].

(1) $$x_{\mathrm{norm}}=x/\max|x|$$

After normalization, each 5 s window contains 1280 samples and serves as input to the beat sequence construction module in CFF-DP. The window stride is set according to the LOSO and personalized fine-tuning protocols.

### 2.4. CFF-DP

**Figure 3:**
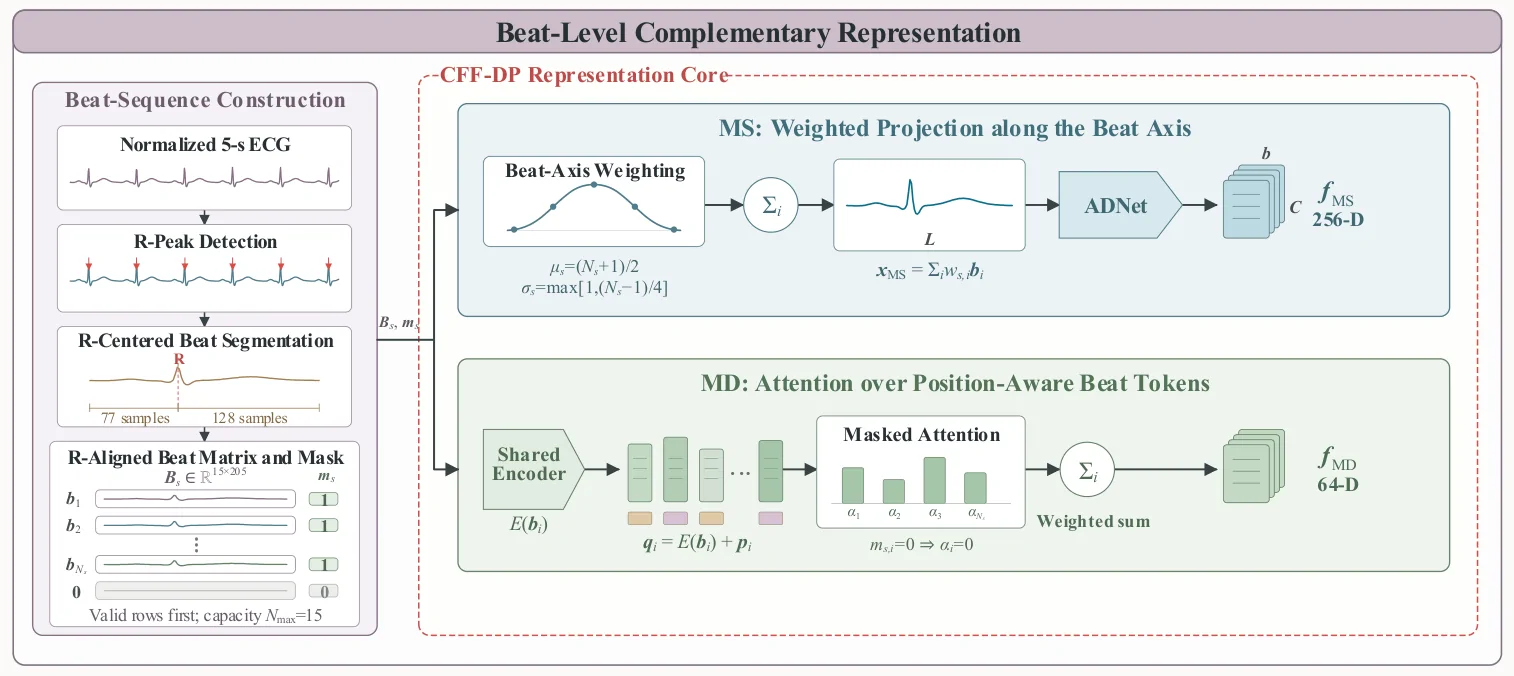
Beat sequence construction and dual-path input organization. The MS path extracts morphology-stable features, and the MD path extracts morphology-difference features. The ellipses denote additional beats and their corresponding representations.

Directly averaging the beats within a window enhances repeated morphology but smooths beat-wise differences. Beat-wise modeling retains local variations but is susceptible to noise in individual beats. Based on this complementary relationship, the beat matrix and valid-position mask produced by the beat sequence construction module are passed to two independent paths, as shown in Figure 3. The MS path aggregates valid beats into a representative beat of length 205 and extracts a 256-dimensional common-morphology feature using ADNet. The MD path independently processes each valid beat with a shared encoder and, after adding sinusoidal positional encoding, obtains a 64-dimensional window representation through masked additive attention.

The MD input consists of R-peak-aligned beats, relative positions, and a valid-position mask; its difference information comes from beat-wise morphology and order retained before aggregation. The two paths share the R-peak positions and valid-beat mask in the input but have independent network parameters. After dual-path feature extraction, the fusion stage projects $f_{\mathrm{MS}}$ and $f_{\mathrm{MD}}$ into a common 320-dimensional space. The GMU generates element-wise gates from the two inputs to form an initial fused representation. A 320 → 256 → 320 residual MLP nonlinearly corrects the gated result, a 320 → 64 → 64 fusion MLP generates window features, and the classification head maps 64 → 32 → 3 to output logits for Baseline, Stress, and Amusement. The MS path describes repeated morphology within the window, while the MD path retains beat-wise encoding before aggregation. Sample-dependent gating combines them into a complementary window-level representation. Figure 3 shows the connection between beat construction and dual-path representation. In the figure, $N_{\max}$ denotes the 15-beat storage capacity, b denotes the batch dimension, and C and L denote the feature channel count and sequence length, respectively.

#### 2.4.1. ECG Beat Sequence Construction

To organize network inputs at the beat level, we detect R peaks in each normalized window using the Pan–Tompkins algorithm [30] and extract beats anchored at the R peaks. Since the P wave precedes the R peak, whereas the ST segment and T wave follow it, the extraction range needs to cover the main waveforms on both sides and retain a longer post-peak interval for repolarization. Wagner et al. used an extraction range of 300 ms before and 500 ms after the R peak for R-peak alignment and representative beat analysis [31]. We use the same asymmetric window of approximately 0.80 s, taking 77 samples before the R peak and 128 samples starting at the R peak at 256 Hz, for a total of 205 samples, to retain the P–QRS–T structure for subsequent morphological encoding. Portions extending beyond the 5 s window boundaries are zero-padded.

Because the number of valid beats varies across 5 s windows, batch training requires a uniform storage capacity along the beat dimension and a mask to record each window’s actual length. We set this capacity to 15 to accommodate the valid beats in the included windows and maintain consistent tensor shapes within a batch. Windows with fewer than 15 beats are zero-padded, and aggregation and beat-wise encoding use only valid beats. A count of 15 beats in 5 s corresponds to approximately 180 beats/min and describes the input capacity; R-peak detection in each window determines how many beats actually enter the computation.

Let $N_{s}$ valid beats be detected in window s and arranged in R-peak order as ${\left\{b_{s,i}\right\}}_{i=1}^{N_{s}}$, where $b_{s,i}\in {\mathbb{R}}^{205}$. The fixed-capacity matrix of R-peak-aligned beats is denoted by $B_{s}\in {\mathbb{R}}^{15\times 205}$, and the valid-position mask by $m_{s}\in {\left\{0,1\right\}}^{15}$. A mask value of 1 indicates that row i corresponds to a real beat. When $N_{s}<15$, zeros are appended to the sequence, when $N_{s}>15$, the first 15 beats are retained in temporal order, and subsequent calculations use the retained valid-beat count. Both the representative beat weights and MD attention weights are normalized over valid positions, and padded positions do not participate in feature computation.

#### 2.4.2. ADConv

**Figure 4:**
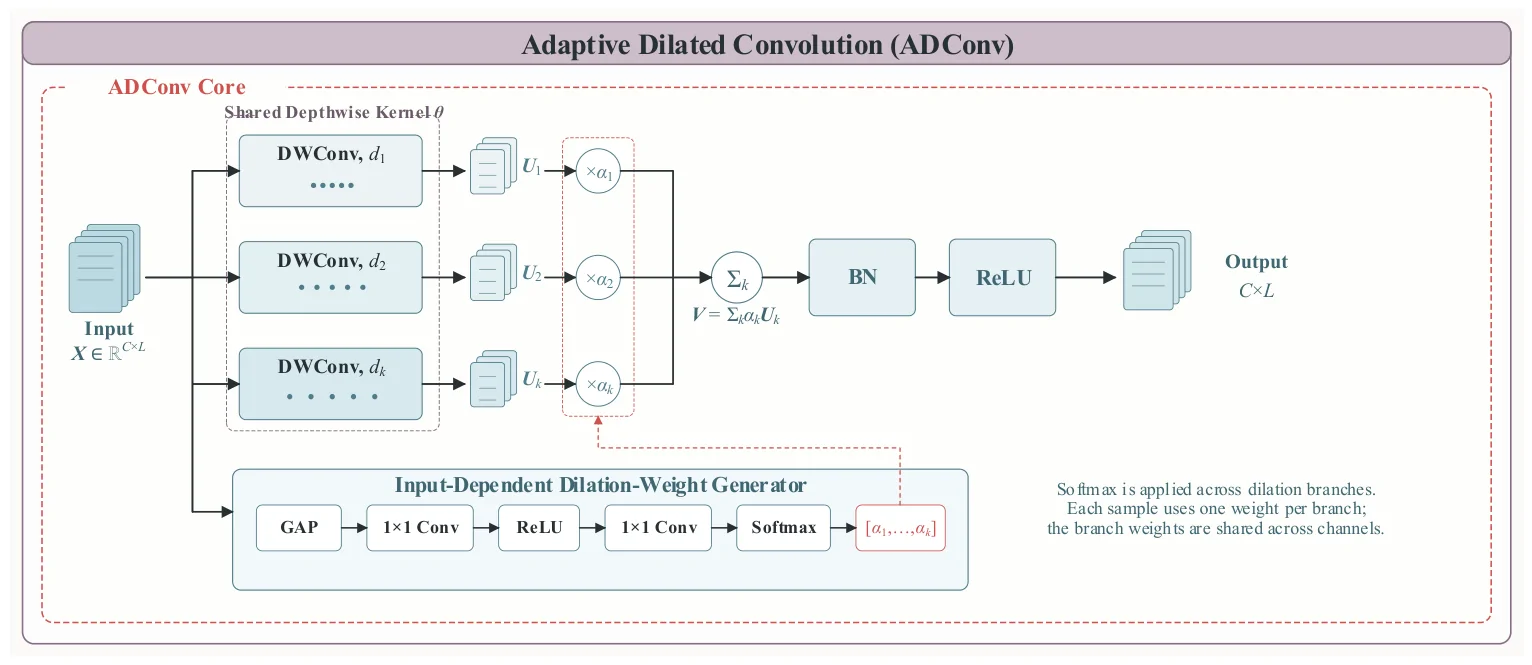
Structure of ADConv. Branches with multiple dilation rates are fused using input-dependent weights. The dots schematically illustrate kernel sampling positions at different dilation rates.

The P wave, QRS complex, and T wave have different temporal scales, making it difficult for a single convolutional receptive field to extract both sharp local changes and broad morphology. ADConv, therefore, uses multiple dilation rates within the same layer and assigns scale weights according to the current input, as shown in Figure 4. Given input features X, the same set of depthwise kernels operates on each dilated sampling grid to obtain branch responses $U_{k}$. A weight generator calculates scale coefficients $\alpha_{k}$, which are used to form a weighted sum of the branch responses. ADConv serves as the depthwise feature extraction unit in ADBlock for multiscale encoding in the MS path.

Let $X\in {\mathbb{R}}^{C\times L}$, where C and L are the channel count and sequence length, respectively. The shared depthwise kernel is $\theta \in {\mathbb{R}}^{C\times 1\times q}$, q is the kernel length, and the dilation rate of branch k is $d_{k}$. Equation (2) gives the response $U_{k}$ of dilated branch k. The K branches reuse one set of C×1×q morphology-detection parameters, and the dilated sampling grid determines the effective receptive field, where DWConv1D denotes one-dimensional depthwise convolution. Compared with structures assigning independent kernels to each scale, the number of depthwise kernel parameters in this design does not increase linearly with K.

(2) $$U_{k}=\mathrm{DWConv}1D(X;\theta ,d_{k})$$

The weight generator performs global average pooling (GAP) on X along the temporal dimension. The first 1 × 1 convolution reduces the channel count to one-eighth of the input channels, rounded down; after a rectified linear unit (ReLU), the second 1 × 1 convolution produces K scale scores. Softmax generates sample-dependent weights $\alpha_{k}$ along the branch dimension, with weighted fusion defined in Equation (3). Branch weights for the same sample are shared across channels. In Equation (3), V denotes the fused output after weighting the dilated branches. Batch normalization (BN) and ReLU are applied sequentially to V to obtain the ADConv output. ADConv is related to the input-conditioned selection used in SKNet, CondConv, and Dynamic Convolution [15,16,17]. Its input dependence acts on the allocation of dilation scales, while shared depthwise kernels ensure that each scale uses the same morphology-detection parameters. With a kernel length of 3, dilation rates of 1, 2, and 4 correspond to nominal receptive fields of 3, 5, and 9 samples. Equation (4) gives the normalization constraint on the scale weights.

(3) $$V=\sum_{k=1}^{K}\alpha_{k}U_{k}$$

(4) $$\sum_{k=1}^{K}\alpha_{k}=1$$

ADConv is embedded as a depthwise feature extraction unit in the one-dimensional inverted residual block ADBlock. The block comprises a 1 × 1 expansion convolution, ADConv, channel recalibration through a lightweight squeeze-and-excitation module (MicroSE), a 1 × 1 projection convolution, and a residual connection. ADNet stacks ADBlocks to form the MS feature extraction network, whose layer configuration is provided in the MS path description.

#### 2.4.3. Morphology-Stable Path

Since individual beats can be affected by local fluctuations, truncation at window boundaries, and occasional noise, the MS path aggregates the valid beats from the beat construction module with weights to extract morphology that recurs within the window [32,33]. To reduce the influence of edge beats on the representative waveform, the weights decrease from the sequence center toward both ends. The Gaussian center $\mu_{s}$ and scale $\sigma_{s}$ depend on the valid-beat count $N_{s}$ to accommodate windows with different numbers of beats, as defined in Equations (5) and (6).

(5) $$\mu_{s}=(N_{s}+1)/2$$

(6) $$\sigma_{s}=\max[1,(N_{s}-1)/4]$$

(7) $$w_{i}=\frac{\exp[-{\left(i-\mu_{s}\right)}^{2}/(2\sigma_{s}^{2})]}{\sum_{j=1}^{N_{s}}\exp[-{\left(j-\mu_{s}\right)}^{2}/(2\sigma_{s}^{2})]}$$

Equation (7) normalizes the Gaussian values at valid positions into beat weights, where i and j are beat indices, $N_{s}$ is the valid-beat count in the current window, and $w_{i}$ is the normalized weight. The scale is lower-bounded by 1 to avoid excessive weight concentration in short sequences. When the valid-beat count exceeds 5, the scale increases with sequence length, keeping the first and last beats two Gaussian standard deviations from the center. Here, adaptation means determining the center and scale from the valid-beat count, rather than learning weights through the network or estimating them from waveform quality. The Gaussian kernel provides smooth nonnegative weights at discrete positions [34], and normalization covers only valid beats. The ablation experiments use uniform aggregation as a control to examine the effect of this center-weighted input on recognition results.

(8) $$x_{\mathrm{MS}}=\sum_{i=1}^{N_{s}}w_{i}b_{i}$$

**Figure 5:**
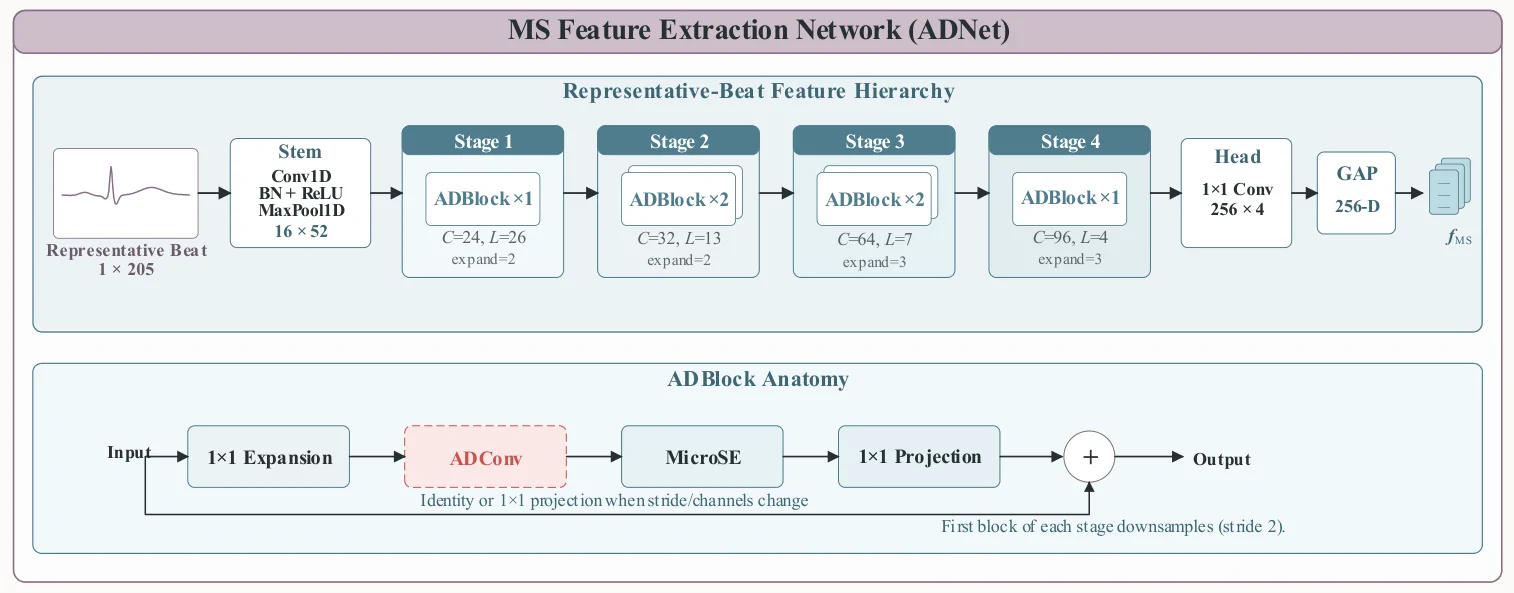
Architecture of the ADConv-based MS feature extraction network. ADBlock denotes an adaptive dilated block, and MicroSE denotes a lightweight squeeze-and-excitation module.

Equation (8) defines the representative beat $x_{\mathrm{MS}}$, where $b_{i}$ denotes valid beat i in the current window and $w_{i}$ is its normalized weight. This aggregation retains repeated P–QRS–T morphology within the window, and $x_{\mathrm{MS}}$ serves as the ADNet input. The MD path instead receives the valid-beat sequence before aggregation. Figure 5 shows feature extraction after the representative beat enters ADNet.

As shown in Figure 5, ADNet extracts features ranging from local waveforms to higher-level morphology from the representative beat. A stem convolution and max pooling first reduce temporal resolution, followed by four feature extraction stages. Their output channel counts are 24, 32, 64, and 96, and their ADBlock counts are 1, 2, 2, and 1. Table 2 lists the network configuration at each stage. In *C*, Mid *C*, Out *C*, and Seq *L* denote the input channel count, expanded channel count, output channel count, and sequence length, respectively; s and d denote stride and dilation rate, respectively.

ADBlock uses the inverted residual and linear bottleneck structure of MobileNetV2 [35], sequentially performing 1 × 1 expansion convolution, ADConv, MicroSE channel recalibration, and 1 × 1 projection convolution, followed by addition to the shortcut branch. When the stride or channel count changes, the shortcut uses a 1 × 1 convolution and BN to match dimensions. MicroSE follows the squeeze-and-excitation (SE) mechanism [36]: global average pooling is first applied along the temporal dimension, and two bias-free fully connected mappings then generate channel weights. The bottleneck channel count is the input channel count divided by 16 and rounded down, with a minimum of 4. The two mappings use ReLU and Hardsigmoid activations, respectively, and the resulting weights are multiplied with the input features channel by channel. The head maps the feature map to 256 channels, and global average pooling produces a 256-dimensional MS feature.

To match multiscale sampling to the temporal resolution of the feature sequence, non-downsampling ADBlocks use the dilation set {1, 2, 4} in the second stage and {1, 2} in the third stage. The first downsampling block in each stage uses a dilation rate of 1, with stride changes performed by the projection convolution.

#### 2.4.4. Morphology-Difference Path

**Figure 6:**
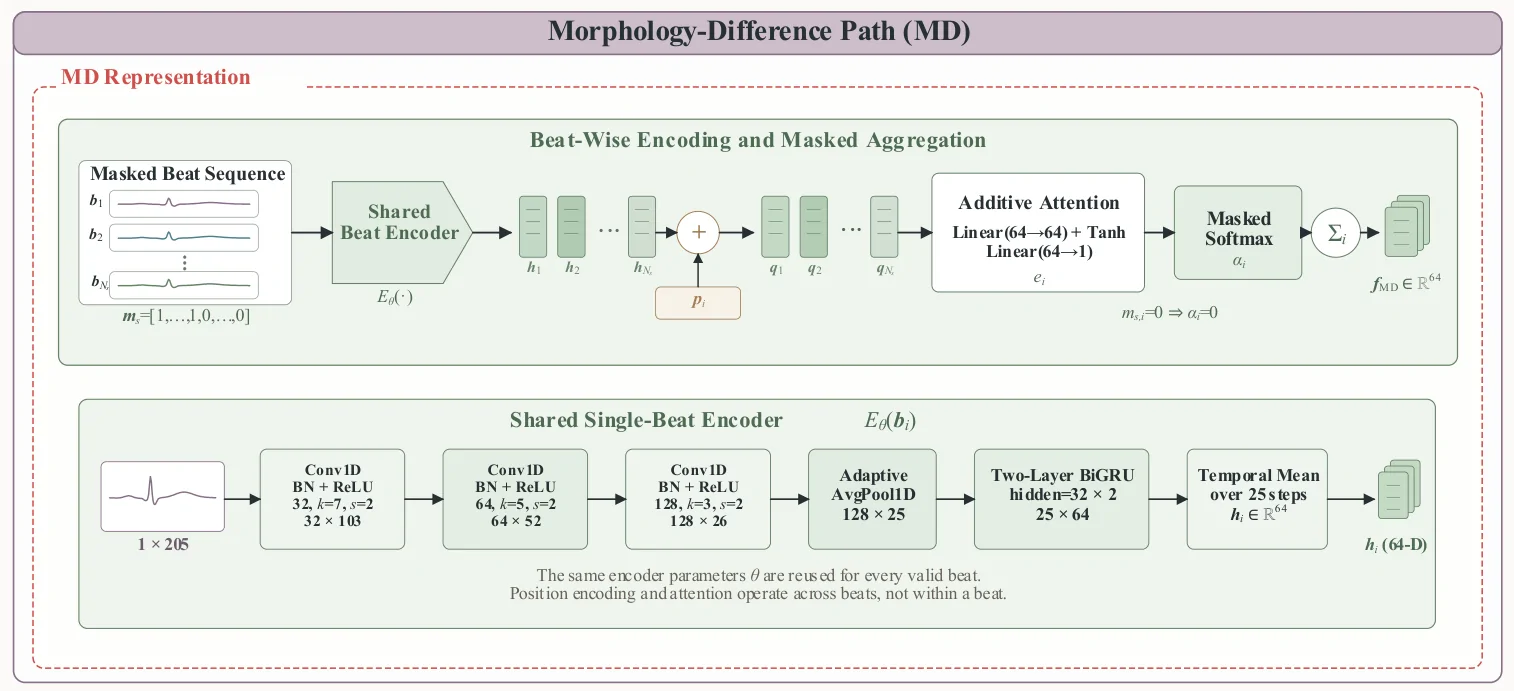
MD path architecture. A shared beat encoder and additive attention are used to aggregate beat features. The ellipses denote additional beats and their corresponding representations.

The representative beat helps extract morphology that recurs within a window, although aggregation smooths the local variations between beats. To retain beat-wise information complementary to the MS path, the MD path independently encodes each valid beat before window-level aggregation, as shown in Figure 6. The same shared encoder processes every beat: three one-dimensional convolutional layers extract local waveforms, and two BiGRU layers learn bidirectional dependencies within a single P–QRS–T cycle [37,38]. Sharing parameters gives each beat position the same morphological encoding rule, while subsequent positional encoding and masked attention retain the relative order between beats.

The three convolutional layers have 32, 64, and 128 output channels, and adaptive average pooling (AAP) sets the feature sequence length to 25. The shared encoder $E_{\theta }(\cdot )$, whose learnable parameters are denoted by θ, encodes beat i into a 64-dimensional vector $h_{i}$ through two BiGRU layers and temporal averaging within the beat. Each BiGRU layer has 32 hidden units per direction. To retain the relative order of beats within the window, we add 64-dimensional sinusoidal positional encoding $p_{i}$ to the beat features to form position-aware representations, as defined in Equations (9) and (10). The positional encoding follows the sinusoidal form used in Transformer [39].

(9) $$h_{i}=\mathrm{Mean}(\mathrm{BiGRU}(\mathrm{AAP}(\mathrm{CNN}(b_{i}))))$$

(10) $$q_{i}=h_{i}+p_{i}$$

To summarize varying numbers of beat encodings into a fixed-dimensional window representation, the MD path uses additive attention [40]. A scoring network with a 64-dimensional hidden layer generates a scalar $e_{i}$ for each position-aware beat vector $q_{i}$. Softmax is calculated only over valid beat positions, and the mask $m_{s,i}$ sets attention weights at padded positions to zero. Equations (11)–(13) define attention scoring and normalization and form the MD feature $f_{\mathrm{MD}}$ as a weighted sum of valid position-aware vectors $q_{i}$.

(11) $$e_{i}=v^{T}\tan h(Wq_{i}+c)$$

(12) $$\alpha_{i}=\frac{m_{i}\exp(e_{i})}{\sum_{j}m_{j}\exp(e_{j})}$$

(13) $$f_{\mathrm{MD}}=\sum_{i}\alpha_{i}q_{i}$$

Here, W, v, c are learnable parameters, $\alpha_{i}$ is the normalized attention weight of valid beat i, and $f_{\mathrm{MD}}\in {\mathbb{R}}^{64}$. The weight $\alpha_{i}$ corresponds to a discrete beat position and represents the relative contribution of each beat encoding to the window-level MD representation. Table 3 provides the MD network structure parameters.

#### 2.4.5. Feature Fusion Layer

The MS and MD paths describe common morphology within the window and beat-wise information before aggregation, respectively, but the two feature types differ in dimension, distribution, and contribution to different samples. Let the feature fusion layer receive the 256-dimensional MS feature $f_{\mathrm{MS}}$ and the 64-dimensional MD feature $f_{\mathrm{MD}}$. Direct concatenation or fixed weighting has difficulty adjusting their relative roles according to the current window. We therefore use a GMU to first project the 256-dimensional MS feature and 64-dimensional MD feature into a common 320-dimensional space through two independent nonlinear mappings and then generate a 320-dimensional element-wise gate z from the concatenation of the original features. The gate controls the relative contributions of the two paths along the feature dimension, allowing fusion weights to vary with the current window. The initial fused representation is $z⊙h_{\mathrm{MS}}+(1-z)⊙h_{\mathrm{MD}}$, where $h_{\mathrm{MS}}$ and $h_{\mathrm{MD}}$ denote the projected features of the two paths after tanh activation, z denotes the gating vector obtained by applying Sigmoid to the concatenated features, ⊙ denotes element-wise multiplication, and 1−z denotes the complementary gate for the MD path. To retain the direct information from gated fusion and learn higher-order interactions between paths, a 320 → 256 → 320 residual MLP transforms the initial representation and adds it to the original GMU output. A 320 → 64 → 64 fusion MLP then uses layer normalization (LN) and nonlinear mappings to compress it into a 64-dimensional window representation. The classification head maps 64 → 32 → 3 through fully connected layers to output logits for Baseline, Stress, and Amusement. Table 4 lists the layer configurations for projection, gating, residual mapping, fusion mapping, and classification.

### 2.5. Deviation-Based Beat-Oriented Augmentation (DBOA)

Since bandpass filtering alone has difficulty removing in-band ECG artifacts caused by muscle activity and electrode motion, DBOA constructs training samples with ma, em, and their mixture from NSTDB [25]. Augmentation needs to introduce noise variation while retaining recognizable beat morphology. In research on wearable ECG quality, the SNR boundaries for reliable QRS detection and full waveform analysis are approximately 5 dB and 18 dB, respectively [41], providing a task-related context for setting noise strength. We use a continuous scheduling range of 10–30 dB and compare neighboring ranges in the parameter sensitivity analysis to examine its suitability on the validation set.

Within a window, some beats deviate more from the representative morphology than others, so the same perturbation applied to all beats may obscure their existing local differences. DBOA therefore first calculates the normalized morphological deviation $r_{i}$ of valid beat i, denoted by $b_{i}$, from the representative beat $x_{\mathrm{MS}}$, and then determines the target SNR $\gamma_{i}$ and augmentation probability $\rho_{i}$ from this deviation. For a smaller deviation, the augmentation probability is higher and the target SNR is lower; for a larger deviation, augmentation is less likely and the noise is weaker. Equation (14) calculates normalized morphological deviation, Equation (15) determines target SNR from the deviation, Equation (16) determines augmentation probability, and Equation (17) defines Bernoulli sampling. Morphological deviation lies in [0, 1]. The lower and upper bounds of target SNR are $\gamma_{\min}=10\;\mathrm{dB}$ and $\gamma_{\max}=30\;\mathrm{dB}$, respectively; the upper and lower bounds of augmentation probability are $\rho_{\max}=1$ and $\rho_{\min}=0$, respectively; and the numerical stability term is $\varepsilon =10^{-12}$. DBOA acts only on valid beats in the MD path during training, while the representative beat in the MS path remains unchanged.

(14) $$r_{i}=\mathrm{clip}\left(\frac{{\left\|b_{i}-x_{\mathrm{MS}}\right\|}_{2}}{{\left\|b_{i}\right\|}_{2}+{\left\|x_{\mathrm{MS}}\right\|}_{2}+\varepsilon },0,1\right)$$

(15) $$\gamma_{i}=\gamma_{\min}+(\gamma_{\max}-\gamma_{\min})r_{i}$$

(16) $$\rho_{i}=\rho_{\max}-(\rho_{\max}-\rho_{\min})r_{i}$$

(17) $$z_{i}∼\mathrm{Bernoulli}(\rho_{i})$$

Algorithm 1 gives the beat-wise deviation calculation, Bernoulli sampling, noise scaling, and mask handling procedures of DBOA.
**Algorithm 1.** Pseudocode for deviation-based beat-oriented augmentation (DBOA).**Input:** Beat tensor $B=\{b_{s,i}\}$; representative beats $X=\{x_{s}\}$; valid-beat mask $M=\{m_{s,i}\}$; resampled NSTDB records $N_{\mathrm{ma}}$ and $N_{\mathrm{em}}$ of lengths $T_{\mathrm{ma}}$ and $T_{\mathrm{em}}$; beat length L=205; $\gamma_{\min}=10\;\mathrm{dB}$; $\gamma_{\max}=30\;\mathrm{dB}$; $\rho_{\max}=1$; $\rho_{\min}=0$; $\varepsilon =10^{-12}$. **Output:** Augmented beat tensor $\tilde{B}$; representative beats X remain unchanged. 1: for each beat $b_{s,i}$ with $m_{s,i}=1$ do   2: $r_{s,i}\;\leftarrow \;\mathrm{clip}(\|b_{s,i}-x_{s}{\|}_{2}/(\|b_{s,i}{\|}_{2}+\|x_{s}{\|}_{2}+\varepsilon ),\;0,\;1)$   3: $\gamma_{s,i}\;\leftarrow \;\gamma_{\min}+(\gamma_{\max}-\gamma_{\min})r_{s,i}$   4: $\rho_{s,i}\;\leftarrow \;\rho_{\max}-(\rho_{\max}-\rho_{\min})r_{s,i}$   5: $z_{s,i}∼\mathrm{Bernoulli}(\rho_{s,i})$   6: if $z_{s,i}=1$ then      7: c∼Uniform({ma, em, ma+em})      8: h∼Uniform({1,2}); $\tau ∼\mathrm{UniformInteger}(0,\;\min(T_{\mathrm{ma}},T_{\mathrm{em}})-L)$      9: n←segment(N(c),h,τ,L); if c=ma+em, n←segment(N(ma),h,τ,L)+segment(N(em),h,τ,L)      10: n←n−mean(n)      11: $a\leftarrow \sqrt{\frac{P(b_{s,i})}{P(n)10^{\gamma_{s,i}/10}+\varepsilon }}$, where $P(v)=L^{-1}\sum_{t}v_{t}^{2}$      12: ${\tilde{b}}_{s,i}\leftarrow b_{s,i}+an$   13: else      14: ${\tilde{b}}_{s,i}\leftarrow b_{s,i}$   15: end if 16: end for 17: Keep all padded positions ($m_{s,i}=0$) unchanged and return $\tilde{B}$.

To give the noise the same temporal resolution as the ECG beats, NSTDB recordings are first resampled to 256 Hz. For each valid beat, one of ma, em, and ma + em is selected with equal probability, and a 205-sample noise segment is extracted using a random channel and starting position. Mixed noise is the sum of the two noise types at the same channel and starting position. Let $n_{i}$ be the mean-centered noise segment. To adjust its amplitude according to target SNR, Equation (18) first calculates the mean-square power of the signal v with length L.

(18) $$P(v)=L^{-1}\sum_{t}v_{t}^{2}$$

On this basis, Equations (19) and (20) use the power ratio to obtain the noise scaling factor $a_{i}$ and construct the augmented beat ${\tilde{b}}_{i}$. Scaled noise is added when the Bernoulli variable is 1, and the original beat is retained when it is 0. The augmented signal retains the original state label. The numerical stability term prevents unstable division when a norm or the noise power approaches zero.

(19) $$a_{i}=\sqrt{\frac{P(b_{i})}{P(n_{i})10^{\gamma_{i}/10}+\varepsilon }}$$

(20) $${\tilde{b}}_{i}=b_{i}+z_{i}a_{i}n_{i}$$

### 2.6. Experimental Settings and Evaluation Protocols

The models are implemented in PyTorch 2.4.1, and training and inference efficiency tests are performed on an NVIDIA GeForce RTX 4090 D GPU. To examine cross-subject recognition and target-subject calibration, we follow the idea of LOSO pretraining and short-term subject fine-tuning used by the Convolutional Frequency-Attention Network (CFAN) [42]. On this basis, we use a fixed validation cohort, separate from the test cohort, to determine training hyperparameters and select the training epoch. Personalized fine-tuning follows the LOSO optimization settings with additional preset regularization. Nested calibration intervals and a common test interval are used in the personalized stage to compare results under different calibration budgets.

The proposed model uses the AdamW optimizer and weighted cross-entropy loss under both protocols, with class weights calculated from the class frequencies in the respective training sets. The training batch size is 1024, and weight decay is 2 × 10^−5^. Initial learning rates for the MS path, MD path, and fusion and classification components are 2.5 × 10^−4^, 3 × 10^−5^, and 2.5 × 10^−4^, respectively. Learning rates stay at their initial values for the first 5 epochs and then follow cosine decay. DBOA is used during training, and both DBOA and Dropout are disabled during validation and testing. Each test fold is trained and evaluated using five different random seeds.

#### 2.6.1. Subject-Independent Leave-One-Subject-Out Evaluation

To separate training, validation, and test data at the subject level, we use 12-fold LOSO evaluation with a fixed validation cohort. S2, S3, and S4 form the validation cohort for hyperparameter determination and epoch selection; S5–S11 and S13–S17 form the test cohort of 12 participants. In each fold, one participant is designated as the test subject, and the other 11 participants in the test cohort form the training set, while S2–S4 always serve as the validation set. Thus, each fold contains 11 training subjects, 3 validation subjects, and 1 test subject. The three sets are mutually exclusive at the subject level, and the overall results are equally weighted across the 12 test subjects. In the LOSO stage, the training, validation, and test sets all use a 5 s window length and a 5 s stride within each subject’s continuous state recordings, with no overlapping windows within any set; the sets are kept independent through subject-level partitioning. The model is trained for 30 epochs in each fold. Dropout is enabled for the MS path, MD path, and fusion and classification components, with dropout probabilities set to 0.05; the corresponding probability for the residual MLP is set to 0. At each epoch, we calculate Macro-F1 separately for S2, S3, and S4 and select the model by their equally weighted mean, choosing the earliest epoch in the event of a tie. After training, we load the selected weights and evaluate the test subject for that fold once. Each of the 12 folds covers one test subject, and the model is independently initialized and trained with five seeds in each fold. Repeated results for each subject are summarized using the same statistical rules to present both between-subject differences and random-run variability.

#### 2.6.2. Personalized Fine-Tuning Protocol

To examine labeled short-term calibration within the same subject and adjust pretrained representations using limited target data, personalized experiments start from the selected LOSO model for the corresponding test fold and random seed and update all network parameters. Following CFAN’s use of the beginning of each state recording for calibration [42], we use the first 20, 30, or 40 s of the Baseline, Stress, and Amusement recordings as fine-tuning intervals. The 5 s training windows have a stride of 0.5 s, yielding 31, 51, and 71 windows per class for the three durations. The three calibration intervals are nested, and all tests use the portion of each state recording after 45 s. Non-overlapping 5 s test windows continue to the end of the recording, giving the different calibration budgets the same test samples. The gaps between the end of calibration and the start of testing are 25, 15, and 5 s, respectively.

Fine-tuning lasts 60 epochs, with the common optimization settings inherited from LOSO. Because short-term calibration data are limited and overlapping windows share most of their samples, we add regularization by increasing Dropout by 0.05 over the LOSO values for the MS path, MD path, fusion and classification components, and residual MLP. The increment and the number of epochs are fixed before fine-tuning. After training, the epoch-60 weights are used to evaluate the fixed test interval once.

To examine the role of pretraining in short-term personalized recognition, we train a control model from random initialization using only the target subject’s calibration data. This control uses the first 20, 30, or 40 s of each class and the same training settings as fine-tuning the proposed model. Both training approaches are evaluated using the weights at epoch 60, and all three budgets are evaluated on the common test interval after 45 s in each class recording.

#### 2.6.3. Evaluation Metrics and Statistical Analysis

Because the three WESAD classes have unequal sample counts, Macro-F1, which weights the classes equally, is used as the primary evaluation metric, while accuracy (ACC) provides a supplementary measure of overall prediction correctness. The number of classes is C = 3, and N is the total number of test windows. The number of true positives for class c is denoted by ${\mathrm{TP}}_{c}$, while ${\mathrm{FP}}_{c}$ and ${\mathrm{FN}}_{c}$ denote false positives and false negatives, respectively. Equation (21) defines ACC. Equations (22)–(24) define precision $P_{c}$, recall $R_{c}$, and $F1_{c}$ for class c, respectively. Equation (25) defines Macro-F1 as the equally weighted average over the three classes. When a denominator is zero, the corresponding class precision, recall, or F1 is set to 0.

(21) $$\mathrm{ACC}=\frac{\sum_{c=1}^{C}{TP}_{c}}{N}$$

(22) $$P_{c}=\frac{{TP}_{c}}{{TP}_{c}+{FP}_{c}}$$

(23) $$R_{c}=\frac{{TP}_{c}}{{TP}_{c}+{FN}_{c}}$$

(24) $${F1}_{c}=\frac{2P_{c}R_{c}}{P_{c}+R_{c}}$$

(25) $$macro\text{-}F1\;=\;\frac{\sum_{c\;=\;1}^{C}F1_{c}}{C}$$

Macro-F1 and ACC for each test subject are first averaged across five seeds, and overall performance is then calculated as the equally weighted mean across the 12 test subjects. Standard deviation (SD) describes dispersion across subjects and seeds separately: SD(subject) is the sample SD of the 12 subject-level means, and SD(seed) is the sample SD of the five seed-level means, each calculated by equally weighting the subjects. Model differences are assessed using two-sided paired *t*-tests on the five-seed means for the same subjects. The 95% confidence intervals in the performance plots describe uncertainty in estimating the population mean performance across subjects. For key model comparisons and ablation controls, differences between the five-seed means for the same subject are averaged to obtain the mean paired difference, reported in percentage points as an unstandardized effect size. Two-sided *t*-based 95% confidence intervals for the differences are calculated from the paired differences of the 12 subjects. Superscripts a, b, and c in the tables correspond to p<0.001, p<0.01, and p<0.05, respectively; the comparison targets are specified in the accompanying text.

## 3. Results

### 3.1. Main and Comparative Experiments

**Figure 7:**
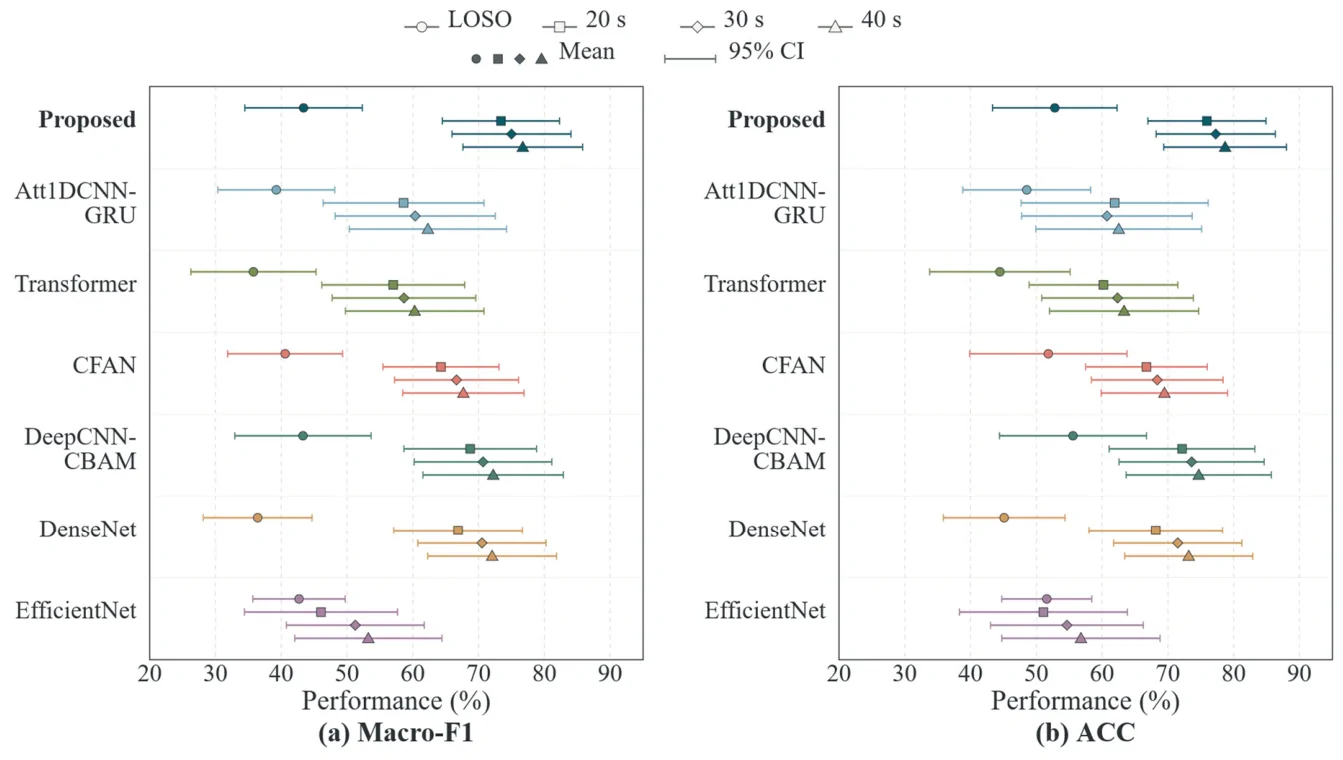
Means and 95% confidence intervals based on subject-level results for seven models under different evaluation settings. (**a**) Macro-F1; (**b**) ACC. LOSO denotes subject-independent evaluation, and 20, 30, and 40 s denote fine-tuning duration per class. Horizontal lines and end caps represent two-sided 95% *t* confidence intervals calculated from subject-level five-seed means.

For cross-subject recognition and short-term personalization, we compare CFF-DP with Att1DCNN-GRU [10], Transformer [39], CFAN [42], DeepCNN-CBAM [43], DenseNet [44], and EfficientNet [45]. They are evaluated under 12-fold LOSO and fine-tuning with 20, 30, and 40 s per class. Structures requiring two-dimensional inputs are converted to one-dimensional implementations along the temporal axis. The personalized experiments provide all models with the same target-subject data, a 60-epoch fine-tuning budget, and a fixed test interval. Each test subject’s metrics are first averaged across five random seeds and then equally weighted across the 12 test subjects. Table 5 reports overall metrics and both dispersion measures, and Figure 7 presents subject-averaged means and 95% confidence intervals. Values are percentages, expressed as the equally weighted subject mean ± SD(subject)/SD(seed), with both SDs calculated as sample standard deviations; 20, 30, and 40 s denote the fine-tuning duration per class. SD(seed) is not applicable to the simple baselines and is shown as —. Repeated random predictions are first averaged within each subject, and all three fine-tuning budgets share the same test interval. Superscripts a, b, and c denote p<0.001, p<0.01, and p<0.05, respectively, for two-sided paired *t*-tests against Proposed using the five-seed means of the 12 subjects. This table reports nominal *p*-values.

Both the proposed model and the six comparison models selected configurations by the equally weighted mean Macro-F1 of fixed validation subjects S2–S4; test subjects were excluded. With learning rates and weight decay fixed for each model, all seven used the same manual Dropout candidate comparison. Supplementary Table S1 reports candidate values, configuration counts, and selected settings for the six comparison models. The selected configurations may still be optimized.

To compare prediction methods that do not use ECG features, we use two simple baselines: always predicting the majority class, Baseline, and randomly predicting the three classes with equal probabilities. Both are evaluated on the corresponding LOSO test windows and the common personalized test windows.

Under LOSO, CFF-DP achieved 43.39% Macro-F1 and 52.81% ACC. Compared with DeepCNN-CBAM, its Macro-F1 differed by 0.09 percentage points, while its ACC was 2.76 percentage points lower. With differences calculated as CFF-DP minus DeepCNN-CBAM, the 95% confidence intervals for the Macro-F1 and ACC differences were approximately −5.00 to 5.18 and −8.74 to 3.22 percentage points, respectively; neither difference was significant. Macro-F1 weights the F1 values of the three classes equally, whereas classes with more samples have a greater influence on ACC within each subject. These means indicate similar class-balanced performance, with lower overall accuracy for CFF-DP. Among the other models, EfficientNet and CFAN had LOSO Macro-F1 values of 42.70% and 40.59%, respectively, with smaller gaps from the proposed method than Att1DCNN-GRU, Transformer, and DenseNet. CFF-DP improved Macro-F1 by 7.62 and 6.98 percentage points over Transformer and DenseNet, respectively, both with p<0.05, showing differences among window encoding methods under class-balanced evaluation.

On the same LOSO test windows, always predicting Baseline and uniform random prediction achieved Macro-F1 values of 23.06% and 31.62%, respectively, both below CFF-DP. However, always predicting Baseline achieved an ACC of 52.88%, slightly above CFF-DP. Thus, ACC close to the majority-class baseline does not fully describe class recognition performance and needs to be considered alongside the primary metric, Macro-F1, and the per-class results.

For personalized fine-tuning using target-subject data from the same recording, the three calibration budgets share fixed test windows to compare overall performance under different short calibration budgets. As calibration increased from 20 s to 40 s per class, CFF-DP’s Macro-F1 increased from 73.39% to 76.72%, and ACC increased from 75.93% to 78.69%. Among the six comparison models, DeepCNN-CBAM performed best at all three durations, while CFF-DP exceeded it by 4.33–4.67 percentage points in Macro-F1 and 3.66–4.00 percentage points in ACC. DenseNet continued to improve with longer fine-tuning durations, reaching a Macro-F1 of 72.05% at 40 s, close to DeepCNN-CBAM’s 72.22%. Transformers and EfficientNet also benefited from calibration, but their Macro-F1 values at 40 s remained below those of the above convolutional models. Both metrics for CFAN and Macro-F1 for Att1DCNN-GRU increased with calibration duration, whereas Att1DCNN-GRU’s ACC decreased from 20 to 30 s and increased at 40 s, indicating that longer calibration does not guarantee monotonic improvement in every metric. In contrast, CFF-DP had the highest mean Macro-F1 and ACC under all three calibration budgets, reflecting its personalized recognition performance in this setting.

After fine-tuning with 20, 30, and 40 s per class, the confidence intervals for the Macro-F1 differences between CFF-DP and DeepCNN-CBAM were approximately 0.02 to 9.32, 0.56 to 8.09, and 0.81 to 8.19 percentage points, respectively. All three intervals were above zero, and the corresponding advantages reached p<0.05, whereas the ACC intervals were approximately −1.51 to 9.08, −0.71 to 8.04, and −0.20 to 8.21 percentage points, respectively. All included zero, and the differences did not reach the 0.05 threshold. Thus, differences from this comparison model under personalization were mainly reflected in class-balanced Macro-F1.

**Figure 8:**
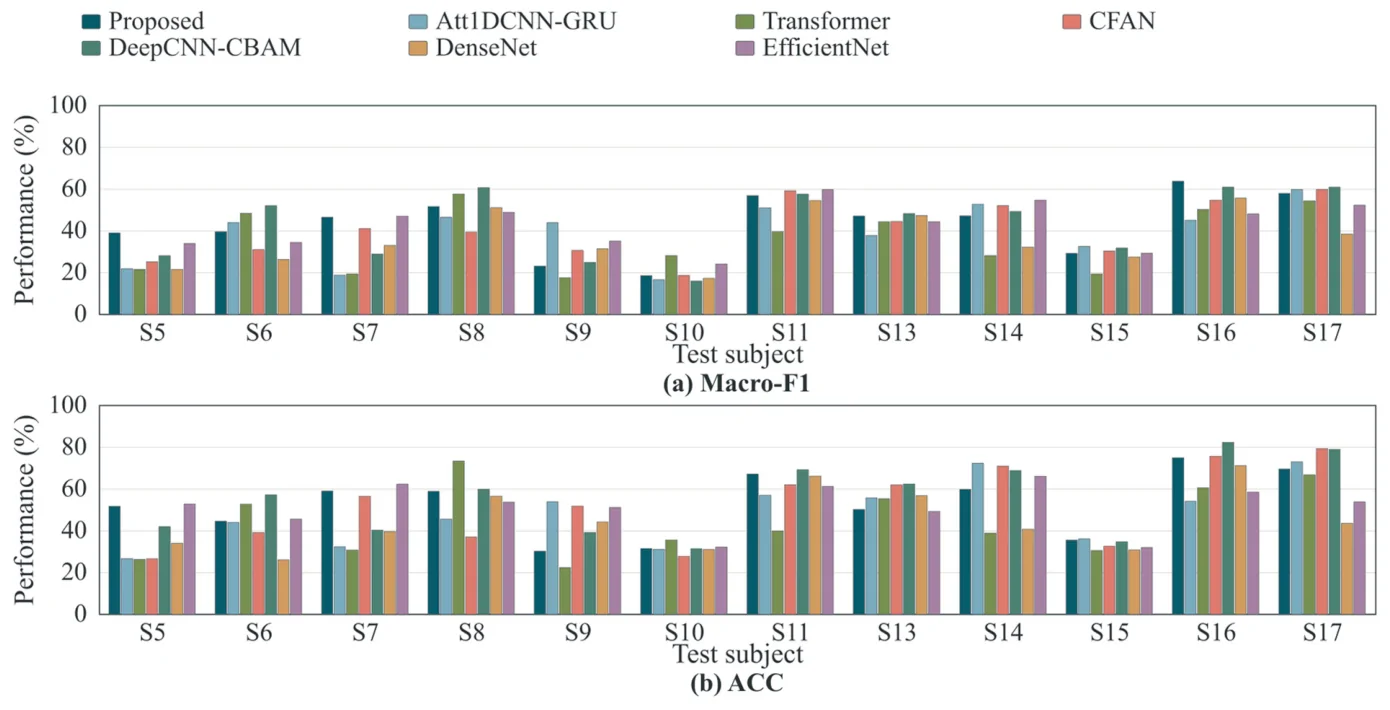
Subject-wise results of the proposed model and six comparison models under 12-fold LOSO. (**a**) Macro-F1; (**b**) ACC. Each bar represents the mean across five random seeds for the corresponding test subject.

**Figure 9:**
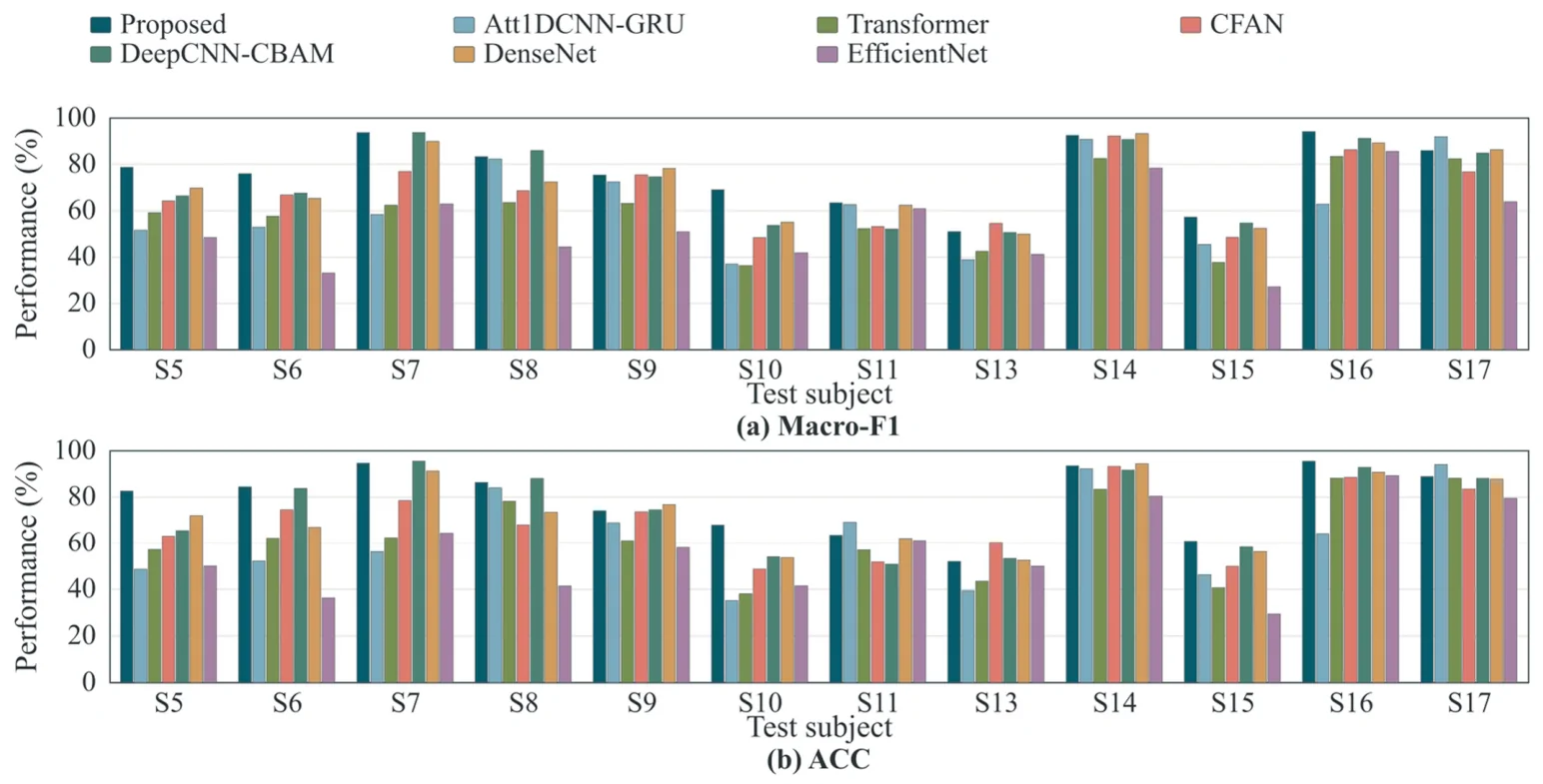
Subject-wise results of the proposed model and six comparison models after personalized fine-tuning with 40 s per class. (**a**) Macro-F1; (**b**) ACC. Each bar represents the mean across five random seeds for the corresponding test subject.

To present performance across subjects and variability caused by random initialization, Figure 8 and Figure 9 show subject-wise results under LOSO and 40 s fine-tuning, respectively; Table 5 also reports SD(subject) and SD(seed).

Across LOSO and the 20, 30, and 40 s fine-tuning settings, CFF-DP’s cross-seed SDs for Macro-F1 and ACC did not exceed 2.13 and 1.98 percentage points, respectively, while cross-subject SDs ranged from 14.01 to 14.90 percentage points. Seed-level averages thus varied less than subject-level means, and substantial between-subject variation remained despite the higher mean performance after fine-tuning.

In addition to between-subject differences, we examine recognition performance for each class. Table 6 lists CFF-DP’s per-class F1 under LOSO and fine-tuning with 40 s per class. Values are percentages, expressed as the equally weighted subject mean ± SD(subject)/SD(seed). Results are first averaged across five seeds within each subject and then equally averaged across the 12 subjects; both SDs are sample standard deviations.

**Figure 10:**
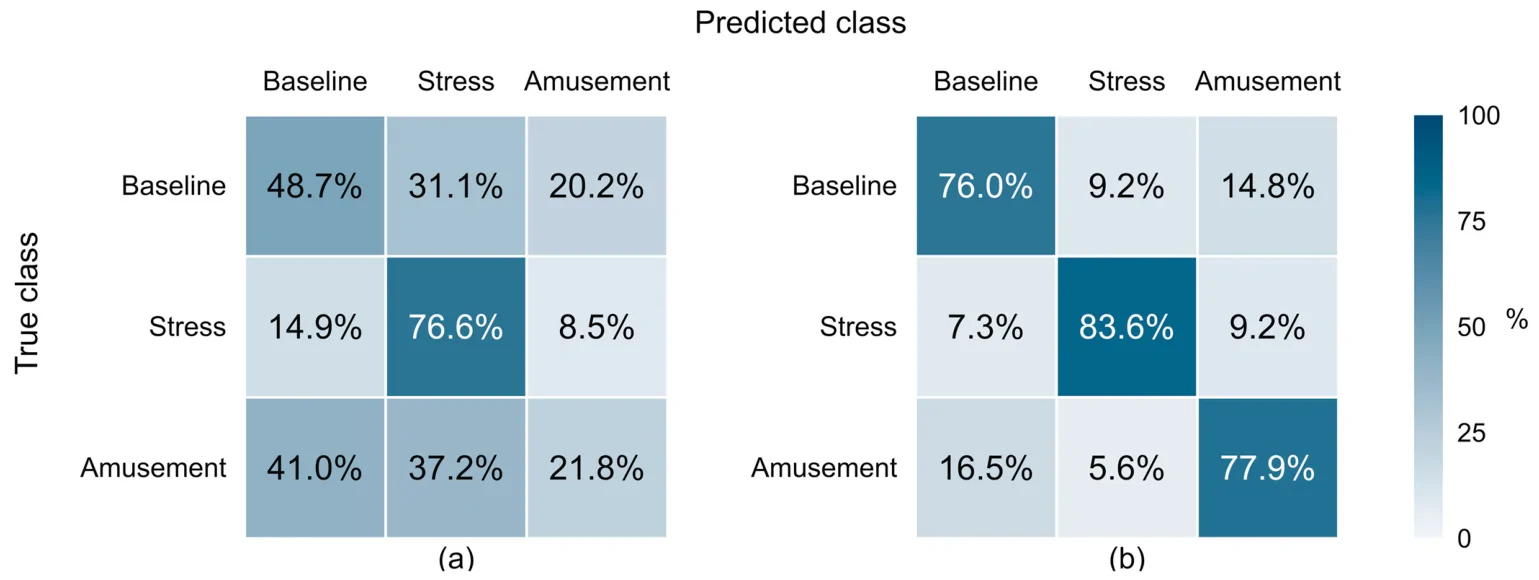
Confusion matrices of CFF-DP on WESAD with equal subject weights. (**a**) LOSO; (**b**) fine-tuning with 40 s per class. Rows represent true classes, and columns represent predicted classes. Each subject–seed matrix is first row-normalized, then averaged across the five seeds and equally averaged across the 12 subjects. Entries are percentages; displayed values in a row may not sum to exactly 100% because of rounding.

Under LOSO, amusement F1 was 16.52%, below its class-specific random baseline, while overall ACC was close to the majority-class baseline. Cross-subject recognition therefore mainly distinguished stress, with limited discrimination of amusement. After fine-tuning with 40 s per class, amusement F1 increased to 66.71% but remained below the other two classes, while stress had the highest F1 under both settings. To illustrate the confusion between classes, Figure 10 shows the prediction distributions for each true class under the two settings.

The two settings use different training conditions and test intervals, reflecting class-level performance for unseen-subject recognition and personalized recognition within the same recording, respectively. Under LOSO, the mean recall for Amusement was only 21.8%; the remaining windows were mainly predicted as Baseline and Stress, at 41.0% and 37.2%, respectively. Confusion between Amusement and the other two classes is therefore a major weakness in the current recognition results. After fine-tuning with 40 s per class, the mean recall for Amusement was 77.9%, and the mean recalls of the three classes were closer.

To examine the role of pretrained initialization in these personalized results, Table 7 compares the proposed model trained from scratch with fine-tuning from the corresponding LOSO weights under three calibration budgets, using the same fixed test interval. Values are percentages, expressed as the equally weighted subject mean ± SD (subject)/SD(seed), with both SDs calculated as sample standard deviations; calibration duration is specified per class. Both training approaches use the weights at epoch 60, and test windows come from the common interval after 45 s in each class recording. The effect size is the mean within-subject difference between fine-tuning after pretraining and training from scratch, expressed in percentage points. Two-sided *t*-based 95% confidence intervals are calculated from the paired differences of the 12 subjects.

Under calibration budgets of 20, 30, and 40 s per class, fine-tuning after pretraining achieved higher Macro-F1 than training from scratch. The mean within-subject differences were approximately 26.2, 28.8, and 30.3 percentage points, with corresponding 95% confidence intervals of 18.8 to 33.6, 21.4 to 36.3, and 23.0 to 37.5 percentage points; all 12 subjects had positive differences under each budget. As calibration duration increased, mean performance from scratch did not increase consistently, whereas mean performance after pretrained fine-tuning increased progressively. Under the current short calibration budgets and training settings, initialization from LOSO pretraining therefore contributes to personalized recognition by the proposed model.

To assess network-level computational cost, Table 8 compares parameter counts, mean single-sample multiply–accumulate operations (MACs), GPU latency at a batch size of 1, and throughput at a batch size of 1024. MACs count multiply–accumulate operations in the network forward pass in eval mode. The proposed model uses mean MACs over real WESAD windows. Latency is measured at a batch size of 1 and reported as mean ± sample SD; throughput uses a batch size of 1024. Both are measured on an RTX 4090 D over 100 timed iterations.

CFF-DP contains 0.6242 M parameters and averages 19.0901 M MACs on real WESAD windows, only 11.7% of EfficientNet’s MACs. Its parameter count is approximately 1/38 of DeepCNN-CBAM’s, and its mean MACs are approximately 1/395.

Shared ADConv kernels avoid duplicating depthwise convolutional parameters across dilation branches, and mask-based beat-wise encoding reduces unnecessary computation at padded positions. At a batch size of 1024, CFF-DP reaches 90,507.0 samples/s, the highest throughput among all models; at a batch size of 1, its 4.870 ms latency ranks fourth. This ranking difference is related to operation granularity and execution: a single-sample forward pass involves beat indexing, dynamic scale weighting, and recurrent encoding, so low MACs do not imply a proportional reduction in scheduling and memory-access costs. Batch processing distributes scheduling costs and increases the number of valid beats processed in parallel on the GPU. The current measurements therefore better reflect throughput advantages for centralized batch processing, while immediate single-window inference still requires model selection based on latency. Att1DCNN-GRU and CFAN have fewer parameters than CFF-DP but higher forward-pass MACs. Although DenseNet and EfficientNet organize parameters differently, their single-window MACs and batch throughput still differ from those of the proposed method. Transformer has lower single-sample latency than CFF-DP but lower batch throughput. Parameter count, MACs, and measured speed therefore need to be considered together with processing batch size.

### 3.2. Ablation Experiments

To examine the roles of dual-path fusion, scale selection, and DBOA, the ablation experiments use the same 12-fold LOSO protocol as the main experiment. M1 retains only the MS path, M2 retains only the MD path, and M3 combines both paths with DBOA disabled. Based on M3, M4 replaces ADConv with depthwise convolution at a single dilation rate, while M5 uses DBOA, allowing the roles of ADConv and DBOA to be examined separately; M5 is the full model. I1 replaces window-adaptive Gaussian aggregation in M3 with uniform weighting. I2 retains the shared kernel and dilation set but replaces input-dependent scale weights with equal weights. I3 replaces the morphology-deviation-based augmentation schedule in M5 with real-noise augmentation at a fixed probability of 0.5, with the target SNR sampled uniformly from 10, 20, and 30 dB.

I4 examines beat construction and input organization by feeding the 1280 samples of the same normalized 5 s window into ADNet in the MS path and CNN–BiGRU in the MD path, in place of the representative beat and beat-wise sequence inputs. Its fusion module, classification head, and parameter capacity remain the same as M3. Another input-organization control, I5, retains beat construction but concatenates valid beats end to end in R-peak order and uses a single CNN–BiGRU to encode the resulting sequence before classification. It is compared with M2, which independently encodes beats before attention aggregation. Augmentation is disabled in M1–M4, I1, I2, I4, and I5; M5 uses DBOA, and I3 uses the fixed augmentation described above. Table 9 summarizes the results for each configuration. Values are percentages, expressed as the equally weighted subject mean ± SD(subject)/SD(seed), with both SDs calculated as sample standard deviations. Superscripts a, b, and c denote p<0.001, p<0.01, and p<0.05, respectively. M1, M2, M4, M5, I1, I2, and I4 are compared with M3; I3 is compared with M5, and I5 with M2. This table reports nominal *p*-values.

Without augmentation, the dual-path model M3 had higher Macro-F1 and ACC than either single-path model. Both differences from the MS-only model M1 reached p<0.01, whereas the means exceeded those of the MD-only model M2 without significant differences. Relative to M1, M3 had mean Macro-F1 and ACC differences of approximately 4.1 and 4.8 percentage points, with 95% confidence intervals of approximately 1.64 to 6.53 and 2.15 to 7.47 percentage points, respectively. Relative to M2, the mean differences were approximately 3.1 and 3.5 percentage points, with corresponding intervals of approximately −4.01 to 10.26 and −4.00 to 10.95 percentage points, consistent with the significance results for these comparisons.

In the single-path comparison, M1 achieved 36.19% Macro-F1 and 46.23% ACC, both below M2’s 37.16% and 47.57%. The MS representation emphasizes repeated morphology through representative-beat aggregation, which also smooths beat-wise variation, whereas MD retains each beat’s morphology and relative order before aggregation. Together with the M3 results, this indicates that the main contribution of the MS path lies in its fusion with the MD path. For input organization, M3 exceeded whole-window input I4 by 1.43 percentage points in Macro-F1, while M2 exceeded beat concatenation I5 by 1.26 percentage points. The Macro-F1 and ACC difference intervals were approximately −4.01 to 6.87 and −5.22 to 8.19 percentage points for M3 versus I4 and −9.82 to 12.32 and −9.74 to 14.14 percentage points for M2 versus I5. All included zero, and none of the paired differences reached p<0.05, so these mean trends are consistent with the design of encoding individual beats before window-level aggregation.

In the controls for the receptive-field scale set and scale-weight allocation, M3 exceeded M4 by 3.43 percentage points in Macro-F1 and 3.45 percentage points in ACC and exceeded I2 by 2.13 and 2.53 percentage points, respectively. The corresponding Macro-F1 and ACC difference intervals were approximately −2.54 to 9.40 and −1.01 to 7.91 percentage points for M3 versus M4 and −3.14 to 7.40 and −3.16 to 8.20 percentage points for M3 versus I2. All four intervals included zero, and none of the paired differences reached p<0.05. Thus, the effect of ADConv on recognition performance follows a trend consistent with its multiscale sampling and input-dependent weighting design. For representative-beat aggregation, Gaussian weights reduce the contribution of edge beats and emphasize repeated morphology near the sequence center. Compared with uniform aggregation I1, M3 achieved gains of 0.82 percentage points in Macro-F1 and 2.14 percentage points in ACC. The corresponding difference intervals were approximately −0.45 to 2.10 and 0.42 to 3.84 percentage points; the ACC interval was above zero, and the difference reached p<0.05. Compared with uniform aggregation, Gaussian aggregation showed a significant difference only in ACC, while the change in the primary metric, Macro-F1, followed a trend consistent with the design of emphasizing repeated morphology.

To examine the effects of real-noise augmentation and its allocation according to morphological deviation, M5 is compared with M3 without augmentation and I3 with fixed augmentation. DBOA assigns perturbation probability and intensity according to beat deviation, providing more augmentation to beats close to the repeated morphology while reducing perturbations to deviating beats. Relative to M3, M5 increased Macro-F1 by 3.11 percentage points, with a difference interval of approximately 0.28 to 5.93 percentage points and a difference reaching p<0.05. ACC increased by 1.76 percentage points, with a difference interval of approximately −1.40 to 4.92 percentage points; this difference was not significant. M5 had the highest mean values for both metrics in this ablation comparison. Relative to fixed augmentation I3, M5 increased Macro-F1 by 1.95 percentage points and ACC by 1.98 percentage points. The corresponding difference intervals were approximately −0.01 to 3.92 and 0.06 to 3.90 percentage points, with a significant difference in ACC at p<0.05. The Macro-F1 improvement of DBOA over fixed augmentation I3 followed a trend consistent with deviation-based augmentation, but the difference was not significant. Without DBOA, M3 had a seed-level Macro-F1 SD of 1.87 percentage points, within the range of the comparison models, whereas the corresponding SD of the full model was 0.65 percentage points, suggesting that its lower seed variability may be associated with data augmentation.

### 3.3. Parameter Sensitivity Experiments

To examine the effect of Gaussian scale settings on representative-beat aggregation, we compare scale floors of 1, 2, and 3 on the fixed validation subjects S2–S4, keeping the other network and training settings unchanged and fixing the DBOA SNR range at 10–30 dB. All three settings retain the valid-beat-count-dependent rule in Equation (6) but replace the scale floor. For each fold, the training epoch is selected using the equally weighted mean Macro-F1 of S2–S4. Validation results across folds are then aggregated within each seed, and the mean and sample SD across five seeds are reported. Table 10 lists the validation results. Values are the mean ± sample SD of the aggregated validation results across five seeds. With each scale floor, the Gaussian scale still varies with the valid-beat count; only results from the fixed validation set S2–S4 are used.

The validation Macro-F1 means differed by at most 0.71 percentage points across the three scale lower bounds, indicating similar average performance and low sensitivity within the examined range.

The same validation subjects S2–S4 were used in every fold, so the validation mean after averaging across folds still represented these three subjects. In the reference configuration with a scale lower bound of 1, the seed-level SDs of S2 and S3 after averaging across 12 folds were 12.60 and 10.14 percentage points, respectively. Their fluctuations offset each other only partly when averaged across the three subjects, yielding a validation-mean SD of 7.69 percentage points. Individual seed-level SDs among the 12 test subjects ranged from 3.49 to 18.86 percentage points, but their changes offset each other when averaged with equal subject weights, yielding a test-mean SD of 0.65 percentage points. The difference in variability between the two cohort means thus relates to cohort composition and the degree of cancellation during aggregation.

To test how sensitive DBOA is to the chosen SNR range, we compare 5–25, 10–30, and 15–35 dB on the fixed validation subjects S2–S4. The ranges have equal widths, and all three settings keep the morphological-deviation schedule for augmentation probability and the same five random seeds. For each seed, we first average the results over S2–S4 with equal subject weights. Table 11 reports the mean and sample SD across the five runs. The main experiment uses the preset range of 10–30 dB; test subjects are not included in this parameter analysis. All three SNR ranges have a width of 20 dB. Values are the mean ± sample SD of the aggregated results across five seeds on the fixed validation subjects S2–S4.

The validation results for the three SNR ranges were close, differing by at most 0.27 percentage points in Macro-F1 and 0.41 percentage points in ACC. The preset range of 10–30 dB gave the highest Macro-F1, and its ACC was 0.01 percentage points short of the highest value. These small shifts within the ranges examined left the overall validation performance at a similar level, so 10–30 dB is retained for the main experiment.

### 3.4. Supplementary Experiments on DREAMER

To examine the applicability of the WESAD training settings to another dataset, we conduct supplementary binary LOSO experiments on valence and arousal in DREAMER. Subjects 1–3 form the fixed validation set, while subjects 4–23 are held out for testing in turn. Each fold uses the other 19 subjects for training, with the three sets mutually exclusive at the subject level. The first-lead ECG recorded during the last 60 s of each video stimulus is divided into non-overlapping 5 s windows and normalized within each window; CFF-DP then uses the same R-peak-aligned beat construction. Separate models are trained for the two affective dimensions. The CFF-DP classifier is adjusted to 64 → 32 → 2, and each task uses weighted cross-entropy with class weights estimated from the training set of the current fold.

Each model retains its hyperparameters and training settings from the WESAD experiments. Training runs for 30 epochs per fold, and the epoch is selected by the equally weighted mean Macro-F1 of the three validation subjects before the held-out test subject is evaluated. Training and evaluation use five different random seeds. Results are first averaged across the five seeds for each test subject and then equally averaged across the 20 subjects. The sample SD of these subject-level means describes dispersion, and models are compared using subject-level two-sided paired *t*-tests. This supplementary experiment evaluates LOSO performance before target-subject fine-tuning, with results presented in Table 12. Macro-F1 and ACC are expressed as percentages. Model results are the mean ± sample SD across 20 subjects, after averaging five seeds within each subject. Random-baseline metrics were first averaged over 10,000 predictions within each subject; both baselines were then summarized as the equally weighted mean and sample SD across the 20 subjects. Bold indicates the highest mean for each metric among the proposed method and the six comparison models. Superscripts a, b, and c denote two-sided paired comparisons with Proposed at p<0.001, p<0.01, and p<0.05, respectively, using nominal *p*-values; the means indicate the direction of each difference.

All 20 test subjects had both LOW and HIGH samples in each task, and subject-level Macro-F1 was calculated as the equally weighted mean of the two class F1 scores. The majority-class baseline always predicted HIGH, whereas the uniform random baseline predicted either class with equal probability. Random predictions were repeated 10,000 times, and their metrics were first averaged within each subject, then across the 20 subjects with equal weights, following the main evaluation protocol. Both baselines used the test windows of the corresponding task.

For valence, the proposed method achieved Macro-F1 and ACC means of 45.47% and 51.81%, respectively, both higher than those of the six comparison models. All paired Macro-F1 differences reach p<0.05; the differences from Att1DCNN-GRU, Transformer, and CFAN reach p<0.001. The ACC advantage is mainly seen in comparisons with Att1DCNN-GRU and EfficientNet, while ACC differences from the other models do not reach p<0.05.

For arousal, EfficientNet had the highest Macro-F1 of 44.30%, and Transformer had the highest ACC of 67.50% among the seven models. CFF-DP achieved 40.17% Macro-F1 and 54.85% ACC, below the respective comparison models, with both differences reaching p<0.01. Its mean Macro-F1 exceeds those of Att1DCNN-GRU and DeepCNN-CBAM, but these differences do not reach p<0.05. The proposed method’s mean arousal Macro-F1 was below Transformer, DenseNet, EfficientNet, and CFAN, ranking fifth among the seven models, so its comparative advantage in valence did not extend to arousal. Overall, Macro-F1 for the proposed method and all six comparison models was below the uniform random baseline in both tasks, indicating limited cross-subject recognition of valence and arousal under the current experimental conditions.

## 4. Discussion

On WESAD, the relationship between the models differs under LOSO and personalized fine-tuning. In cross-subject evaluation, CFF-DP has a Macro-F1 close to that of DeepCNN-CBAM and an ACC 2.76 percentage points lower but uses approximately 1/38 of its parameters and 1/395 of its mean MACs. The dual-path beat-level representation therefore maintains class-balanced recognition close to the high-capacity convolutional model with less network computation. When short recordings from the target subject are used, CFF-DP has the highest Macro-F1 and ACC at all three calibration durations. This suggests that individual calibration can reduce the difference in representation between the fixed population model and the target subject.

Under the 20–40 s per-class calibration budgets in this study, fine-tuning the proposed model from LOSO weights achieved higher Macro-F1 than training from scratch using only target-subject calibration data. This indicates that pretrained initialization is an important component of the current personalized results. For cross-subject recognition, per-class analysis shows that Amusement remains difficult to recognize under LOSO, so further improvements should address its confusion with Baseline and Stress.

In the ablation experiments, MS preserves repeated morphology through aggregation, while MD independently encodes each beat before aggregation to retain beat-wise information. Fusion of the two paths without augmentation exceeded both single-path configurations on both metrics, whereas the MS-only path scored below the MD-only path. This indicates that the main contribution of the MS path lies in its fusion with the MD path. For input organization, comparisons of whole-window input I4 with M3 and beat concatenation I5 with M2 show mean trends consistent with beat-wise organization, but the paired differences for both metrics did not reach p<0.05. The Macro-F1 changes with ADConv and Gaussian aggregation, and the Macro-F1 improvement with DBOA over fixed augmentation followed trends consistent with their designs, although the paired differences were not significant. These components act on beat organization, scale responses, and training-sample variation, jointly forming the window-level representation of the full model.

**Figure 11:**
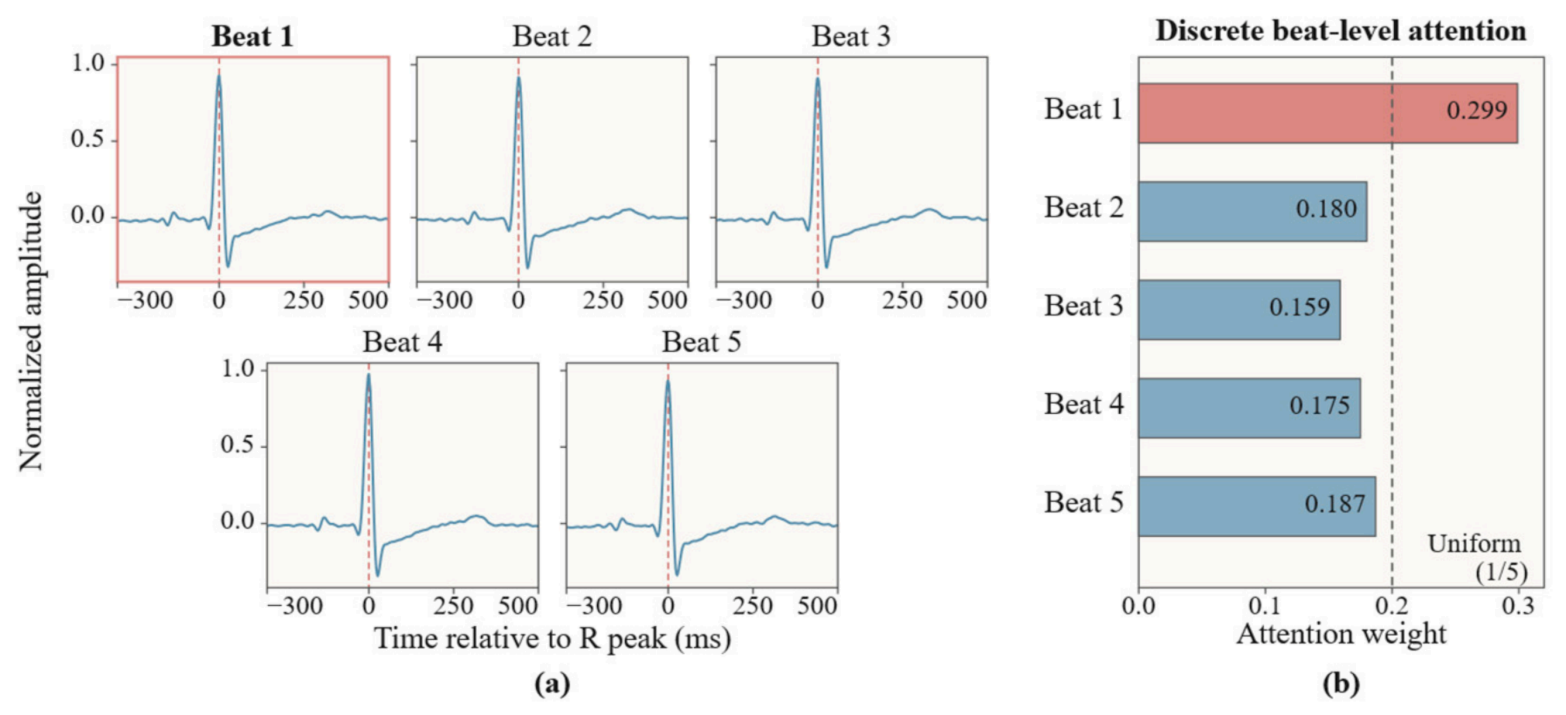
Example of discrete beat-wise attention in the MD path. (**a**) Five normalized R-peak-aligned beats; red dashed lines indicate the R peaks at 0 ms, and the horizontal range is −300 to 500 ms. (**b**) Scalar attention weights for individual beats; the gray dashed line indicates the uniform baseline of 1/5, and coral highlights the largest weight.

Attention in the MD path is calculated at discrete beat positions, with Figure 11 illustrating its allocation over 5 valid beats.

A uniform allocation would give each beat a weight of 0.200. Here, the first beat receives 0.299, and the others receive 0.159–0.187: additive attention weights the beats unequally but keeps every valid beat in the aggregation. The largest weight is approximately 1.88 times the smallest in this example, showing that individual beat representations contribute different proportions to the window summary. Beat-wise morphology encoding and positional information determine this allocation, which selects among beats for aggregation rather than assigning attention to continuous time points inside a beat.

**Figure 12:**
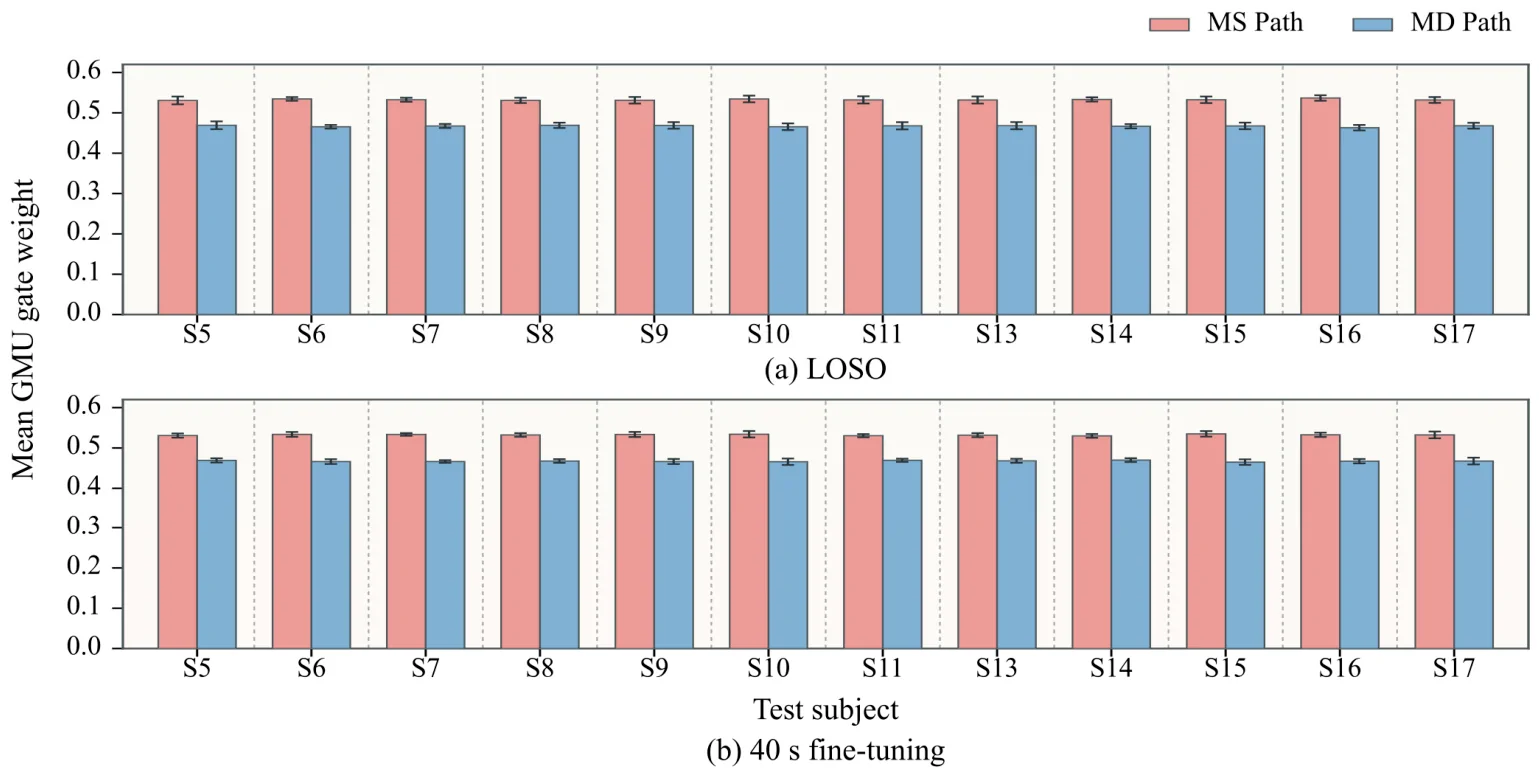
Subject-level GMU gate proportions. (**a**) 12-fold LOSO; (**b**) fine-tuning with 40 s per class. MS and MD denote the morphology-stable and morphology-difference paths, respectively. Bar heights are five-seed averages of means over all test windows and 320 gate dimensions; error bars indicate cross-seed sample SD.

Figure 12 presents the subject-level GMU gate distributions for LOSO and 40 s fine-tuning. The MS path accounts for approximately 0.533 of the overall gate allocation and the MD path for 0.467, with neither path dominating before or after fine-tuning.

These results describe what beat-level complementary representations contribute in the present experiments; where they can be applied also depends on the data source and calibration conditions. WESAD records three states using single-lead ECG in a controlled laboratory setting. The fixed validation cohort is separate from the 12 test subjects, keeping model selection independent of the subjects being tested, while DREAMER adds evaluation with another device and elicitation procedure. Both datasets were acquired under controlled conditions, so generalization in natural activities still requires independent evaluation. For WESAD personalization, calibration uses the initial portion of a recording, and testing uses a fixed interval after 45 s in that same recording, allowing different calibration budgets to be compared on identical test samples. The amount of calibration data and its temporal proximity to testing change together. The current MD path also retains beat-wise morphology and relative order without explicitly taking rhythm information, such as R–R intervals, as input.

Under the present 5 s ECG, subject-independent LOSO setting with WESAD-selected hyperparameters, recognition on both DREAMER tasks remained limited. Future work will examine personalization for both valence and arousal by comparing classifier-only and full-network fine-tuning, using different video stimuli for calibration and testing to examine the role of a small amount of labeled target-subject data. As the current representation mainly describes R-peak-aligned morphology and beat order, rhythm information such as R–R intervals could also be added to test whether joint morphology and rhythm representations improve this task.

## 5. Conclusions

We propose CFF-DP through beat-level complementary feature fusion, with WESAD single-lead ECG three-state classification as the main experiment. Window-adaptive Gaussian weights form a representative beat for the MS path, where ADConv extracts common morphology at multiple scales. The MD path encodes each valid beat independently and uses positional encoding and masked additive attention to form beat-wise representations. A GMU and residual MLP fuse these two types of features. During training, DBOA adjusts the probability of injecting real noise and the target SNR according to morphological deviation. In 12-fold LOSO on WESAD, CFF-DP achieved 43.39% Macro-F1 and 52.81% ACC, with 0.6242 M parameters and an average of 19.0901 M MACs for real WESAD windows. The proposed method had the lowest network MACs among the compared models, although WESAD LOSO recognition mainly distinguished stress, with limited amusement discrimination. With calibration and testing separated by 5 s within the same continuous recording, personalized fine-tuning with 40 s per class achieved 76.72% Macro-F1 and 78.69% ACC, the highest means among the models compared. These results show the advantages of low network computation and personalized recognition within the same recording. The Macro-F1 changes with ADConv and Gaussian aggregation, and the Macro-F1 improvement with DBOA over fixed augmentation followed trends consistent with their designs, although the paired differences were not significant. The training-from-scratch control further shows that initialization from LOSO pretraining contributes to personalized recognition under short calibration budgets. In the supplementary binary DREAMER experiments, the proposed method’s valence Macro-F1 exceeded all six comparison models, while arousal ranked fifth among the seven models. However, all models had Macro-F1 below the uniform random baseline in both tasks, indicating that cross-subject recognition remained limited overall. The limitations remain controlled data acquisition, calibration within the same recording, and efficiency measurements on a GPU. In addition, the current representations mainly describe R-peak-aligned morphology and beat order. Future work will examine target-subject fine-tuning for both valence and arousal on DREAMER and use natural-activity recordings to test generalization across devices and scenarios, add rhythm information such as R–R intervals, and measure end-to-end costs on wearable devices so that performance is evaluated under the corresponding deployment conditions.

## Figures and Tables

**Table 1:** Overview of the WESAD dataset and the three-class task in this study.

Dataset	Subjects	ECG Lead	Sampling Rate	Window	Task Labels	Evaluation
WESAD	15	Chest, single lead	700 Hz → 256 Hz	5 s	Baseline/Stress/Amusement	Fixed validation cohort + 12-fold leave-one-subject-out test

**Table 2:** Structure of the MS feature extraction network.

Stage	Module	In C	Mid C	Out C	SE	Stride	Dilation	Seq L
Stem	Conv1D + MaxPool	1	—	16	No	2, 2	1	205 → 52
1	ADBlock × 1	16	32	24	No	2	1	52 → 26
2	ADBlock × 1	24	48	32	Yes	2	1	26 → 13
2	ADBlock × 1	32	64	32	Yes	1	1/2/4	13
3	ADBlock × 1	32	96	64	Yes	2	1	13 → 7
3	ADBlock × 1	64	192	64	Yes	1	1/2	7
4	ADBlock × 1	64	192	96	Yes	2	1	7 → 4
Head	Conv1D + GAP	96	—	256	No	1	1	4 → 1

**Table 3:** Network structure of the MD path.

Module	Sub-Layer	Input	Output	Kernel/Configuration
Intra-beat	Conv block 1	1 × 205	32 × 103	k=7, s=2
	Conv block 2	32 × 103	64 × 52	k=5, s=2
	Conv block 3	64 × 52	128 × 26	k=3, s=2
	Adaptive pooling	128 × 26	128 × 25	output length: 25
	BiGRU layer 1	25 × 128	25 × 64	hidden: 32 per direction
	BiGRU layer 2	25 × 64	25 × 64	hidden: 32/direction
	Temporal mean	25 × 64	64	mean over beat samples
Output	Positional encoding	64	64	sinusoidal, 64-D
	Additive attention	$N_{s}\times 64$	$N_{s}$	masked Softmax
	Weighted sum	$N_{s}\times 64$	64	valid beats only
	MD feature	64	64	$f_{\mathrm{MD}}$

**Table 4:** Structure of the GMU–MLP feature fusion layer.

Module	Sub-Layer	Input	Output	Activation/Configuration
GMU	MS projection	256	320	tanh
GMU	MD projection	64	320	tanh
GMU	Gate	320	320	sigmoid
GMU	Gated fusion	320 + 320	320	element-wise
Residual MLP	Linear 1	320	256	BN + ReLU
Residual MLP	Linear 2	256	320	BN + ReLU
Residual MLP	Residual add	320 + 320	320	element-wise
Fusion MLP	Linear 1	320	64	LN + ReLU
Fusion MLP	Linear 2	64	64	LN + ReLU
Classifier	Linear 1	64	32	ReLU
Classifier	Linear 2	32	3	logits

**Table 5:** Main and comparative results under the same LOSO and subject-specific fine-tuning protocols.

Model	Metric	LOSO	20 s	30 s	40 s
Proposed	Macro-F1	43.39 ± 14.06/0.65	73.39 ± 14.01/2.13	75.00 ± 14.24/1.70	76.72 ± 14.33/1.79
	ACC	52.81 ± 14.90/0.85	75.93 ± 14.13/1.98	77.27 ± 14.25/1.69	78.69 ± 14.68/1.52
Att1DCNN-GRU [10]	Macro-F1	39.24 ± 13.99/2.38	58.59 ± 19.24/1.68 ^b^	60.36 ± 19.15/1.56 ^b^	62.29 ± 18.81/1.52 ^b^
	ACC	48.54 ± 15.30/3.01	61.90 ± 22.39/0.85 ^c^	60.73 ± 20.40/2.62 ^b^	62.53 ± 19.83/1.92 ^b^
Transformer [39]	Macro-F1	35.77 ± 14.97/3.28 ^c^	57.02 ± 17.10/1.95 ^a^	58.65 ± 17.18/1.11 ^a^	60.28 ± 16.58/2.24 ^a^
	ACC	44.46 ± 16.80/4.29	60.20 ± 17.80/3.22 ^a^	62.34 ± 18.14/1.19 ^a^	63.34 ± 17.84/2.85 ^a^
CFAN [42]	Macro-F1	40.59 ± 13.75/1.81	64.27 ± 13.88/1.92 ^b^	66.64 ± 14.84/1.96 ^b^	67.68 ± 14.51/1.75 ^b^
	ACC	51.82 ± 18.82/1.94	66.74 ± 14.56/1.68 ^b^	68.38 ± 15.75/1.82 ^b^	69.47 ± 15.12/1.64 ^b^
DeepCNN-CBAM [43]	Macro-F1	43.30 ± 16.31/4.65	68.72 ± 15.87/2.57 ^c^	70.67 ± 16.46/2.30 ^c^	72.22 ± 16.80/2.13 ^c^
	ACC	55.57 ± 17.59/4.53	72.15 ± 17.44/2.41	73.61 ± 17.37/2.01	74.69 ± 17.37/2.01
DenseNet [44]	Macro-F1	36.41 ± 13.01/4.08 ^c^	66.88 ± 15.39/1.00 ^b^	70.51 ± 15.35/1.09 ^c^	72.05 ± 15.41/1.03 ^c^
	ACC	45.10 ± 14.56/2.88	68.16 ± 15.98/0.78 ^c^	71.50 ± 15.34/1.24 ^c^	73.17 ± 15.33/1.04 ^c^
EfficientNet [45]	Macro-F1	42.70 ± 11.05/1.76	46.04 ± 18.31/1.68 ^a^	51.25 ± 16.49/1.70 ^a^	53.24 ± 17.60/1.63 ^a^
	ACC	51.59 ± 10.78/2.36	51.08 ± 20.10/4.12 ^a^	54.66 ± 18.27/3.15 ^a^	56.78 ± 18.93/3.25 ^a^
Always Baseline	Macro-F1	23.06 ± 0.23/—	23.41 ± 0.25/—	23.41 ± 0.25/—	23.41 ± 0.25/—
	ACC	52.88 ± 0.81/—	54.11 ± 0.88/—	54.11 ± 0.88/—	54.11 ± 0.88/—
Uniform random	Macro-F1	31.62 ± 0.09/—	31.40 ± 0.10/—	31.40 ± 0.10/—	31.40 ± 0.10/—
	ACC	33.32 ± 0.02/—	33.33 ± 0.02/—	33.33 ± 0.02/—	33.33 ± 0.02/—

**Table 6:** Per-class F1 of CFF-DP on WESAD under LOSO and fine-tuning with 40 s per class.

Class	LOSO F1 (%)	40 s F1 (%)
Baseline	51.08 ± 21.28/1.45	80.47 ± 15.24/2.56
Stress	62.57 ± 20.33/3.50	82.97 ± 17.88/1.53
Amusement	16.52 ± 8.06/4.69	66.71 ± 21.32/1.74

**Table 7:** Comparison of training the proposed model from scratch and fine-tuning after LOSO pretraining.

Calibration	Metric	From Scratch	LOSO + Fine-Tuning
20 s	Macro-F1	47.18 ± 17.01/9.15	73.39 ± 14.01/2.13
	ACC	54.07 ± 15.56/7.88	75.93 ± 14.13/1.98
30 s	Macro-F1	46.16 ± 16.60/8.27	75.00 ± 14.24/1.70
	ACC	53.77 ± 16.21/6.21	77.27 ± 14.25/1.69
40 s	Macro-F1	46.44 ± 16.46/9.76	76.72 ± 14.33/1.79
	ACC	53.37 ± 16.30/8.21	78.69 ± 14.68/1.52

**Table 8:** Model complexity and inference efficiency.

Model	Params (M)	MACs (M)	Latency (ms)	Throughput (Samples/s)
Att1DCNN-GRU [10]	0.5942	504.4641	4.435 ± 0.016	20,048.1
Transformer [39]	4.9380	1567.7034	1.623 ± 0.012	4872.8
CFAN [42]	0.2926	281.3655	0.967 ± 0.009	32,330.5
DeepCNN-CBAM [43]	23.7451	7538.2488	5.490 ± 0.210	3297.4
DenseNet [44]	5.5226	477.3919	8.867 ± 0.296	8709.1
EfficientNet [45]	3.8682	163.6928	4.911 ± 0.190	18,442.3
Proposed	0.6242	19.0901	4.870 ± 0.028	90,507.0

**Table 9:** Ablation results for WESAD three-class classification.

Configuration	Macro-F1	ACC
M1	36.19 ± 13.05/3.53 ^b^	46.23 ± 14.65/6.10 ^b^
M2	37.16 ± 13.70/6.22	47.57 ± 13.66/5.81
M3	40.28 ± 12.75/1.87	51.05 ± 13.91/2.72
M4	36.85 ± 13.32/7.36	47.60 ± 12.27/7.75
M5	43.39 ± 14.06/0.65 ^c^	52.81 ± 14.90/0.85
I1	39.46 ± 11.98/1.73	48.91 ± 12.10/2.14 ^c^
I2	38.15 ± 14.41/6.96	48.52 ± 13.89/5.04
I3	41.44 ± 13.47/2.35	50.83 ± 14.35/2.31 ^c^
I4	38.85 ± 13.81/3.39	49.56 ± 17.32/4.64
I5	35.90 ± 16.52/3.46	45.38 ± 17.25/5.28

**Table 10:** Sensitivity to the Gaussian scale floor on the fixed WESAD validation set.

Scale Floor	Macro-F1 (%)	ACC (%)
1	43.28 ± 7.69	56.36 ± 8.62
2	42.57 ± 6.91	55.19 ± 7.67
3	43.12 ± 6.42	55.15 ± 6.91

**Table 11:** Sensitivity to the SNR range on the fixed WESAD validation set.

SNR Interval	Macro-F1 (%)	ACC (%)
5–25 dB	43.01 ± 7.66	55.96 ± 8.43
10–30 dB	43.28 ± 7.69	56.36 ± 8.62
15–35 dB	43.24 ± 7.41	56.37 ± 8.17

**Table 12:** Supplementary binary LOSO results for valence and arousal on DREAMER.

Model	Valence		Arousal	
	Macro-F1	ACC	Macro-F1	ACC
Att1DCNN-GRU [10]	35.74 ± 7.29 ^a^	44.73 ± 12.56 ^c^	38.33 ± 14.73	53.95 ± 23.25
Transformer [39]	37.91 ± 7.60 ^a^	49.70 ± 12.22	41.82 ± 6.01	**67.50 ± 15.40 ^b^**
CFAN [42]	39.16 ± 5.48 ^a^	48.95 ± 10.33	40.25 ± 9.34	59.92 ± 17.43
DeepCNN-CBAM [43]	40.86 ± 6.33 ^b^	49.58 ± 9.97	39.15 ± 9.34	56.44 ± 18.52
DenseNet [44]	41.76 ± 7.75 ^c^	48.64 ± 11.43	40.97 ± 9.67	59.40 ± 19.56
EfficientNet [45]	42.49 ± 5.78 ^c^	46.53 ± 6.72 ^b^	**44.30 ± 4.83 ^b^**	57.56 ± 8.06
Proposed	**45.47 ± 4.76**	**51.81 ± 6.59**	40.17 ± 5.24	54.85 ± 10.03
Majority class (HIGH)	37.43 ± 5.11	60.83 ± 12.93	41.92 ± 4.92	73.33 ± 14.49
Uniform random	48.49 ± 1.56	50.01 ± 0.03	45.63 ± 4.33	50.01 ± 0.04

## Data Availability

The WESAD data are openly available from the dataset repository at https://archive.ics.uci.edu/dataset/465/wesad+wearable+stress+and+affect+detection (accessed on 26 August 2026). The muscle artifact (ma) and electrode motion (em) noise records used for augmentation are openly available in the MIT-BIH Noise Stress Test Database at https://physionet.org/content/nstdb/1.0.0/ (accessed on 26 August 2026). Access to the DREAMER data can be requested from the providers at https://zenodo.org/records/546113 (accessed on 26 August 2026).

## Supplementary Materials

The following supporting information can be downloaded at: https://www.mdpi.com/article/10.3390/s26196169/s1, Table S1: Dropout candidates and selected settings for the six comparison models.
