# Supplementary material for: ECG-Based Emotion Recognition Using Beat-Level Complementary Feature Fusion
Source: Sensors (Basel). 2026 Sep 29;26(19):6169. doi: 10.3390/s26196169 (PMC13667585; doi:10.3390/s26196169)
Supplement: Supplementary file 1 [file sensors-26-06169-s001.zip › sensors-4589620-supplementary.pdf]

Hyperparameter Settings for the Comparison Models

The six comparison models followed the same manual Dropout comparison procedure and candidate values as the proposed model. Configuration selection used the equally weighted mean Macro-F1 of the fixed WESAD validation subjects S2–S4 under the 12-fold, five-seed protocol; test-subject results were not used.

All six comparison models used a fixed learning rate of 3×10^{-5} and weight decay of 2×10^{-5}. At these fixed settings, Dropout probabilities of 0, 0.05, and 0.10 were evaluated, giving three distinct configurations per model. Table S1 reports the selected Dropout settings.

Table S1. Dropout candidates and selected settings for the six comparison models.

**Model**	**Dropout candidates**	**Evaluated configurations**	**Selected Dropout**
Att1DCNN-GRU	0, 0.05, 0.10	3	0
Transformer	0, 0.05, 0.10	3	0.10
CFAN	0, 0.05, 0.10	3	0
DeepCNN-CBAM	0, 0.05, 0.10	3	0
DenseNet	0, 0.05, 0.10	3	0
EfficientNet	0, 0.05, 0.10	3	0.10
